# Metabolic disturbance in hippocampus and liver of mice: A primary response to imidacloprid exposure

**DOI:** 10.1038/s41598-020-62739-9

**Published:** 2020-03-31

**Authors:** Meilin Zheng, Qizhong Qin, Wenli Zhou, Qin Liu, Shaohua Zeng, Hong Xiao, Qunhua Bai, Jieying Gao

**Affiliations:** 1 School of Public Health and Management, Chongqing Medical University, Chongqing, 400016 P. R. China; 2 Center of Experimental Teaching for Public Health, Experimental Teaching and Management Center, Chongqing Medical University, Chongqing, 401331 P. R. China; 3 China Coal Technology & Engineering Group Chongqing Research Institute, Chongqing, 400039 P. R. China

**Keywords:** Metabolomics, Mass spectrometry, Analytical chemistry, Biochemistry

## Abstract

Imidacloprid (IMI) is one of the most frequently used neonicotinoid insecticides, but recent studies have shown adverse effects on mammals. IMI was found to be neurotoxic and hepatotoxic. In the present study, the effects of repeated oral administration of two doses of IMI (5 and 20 mg/kg/day) for 28 days on hippocampus and liver of female KM mice were studied. The histopathological and biochemical experiments indicated obvious damages to the hippocampus and liver of mice in the high-dose group (20 mg/kg/day). Using a high-throughput metabolomics platform based on ultrahigh performance liquid chromatography/hybrid quadrupole time-of-flight mass spectrometry (UPLC/Q-TOF MS), we studied effects of IMI on metabolic profiles in the hippocampus and liver of mice. Significant differences among the control group, the low-dose group and the high-dose group were clearly presented using multivariate analysis. The changed metabolic profile in the low-dose group (5 mg/kg/day) revealed that the metabolic disturbance in the hippocampus and liver of mice had been induced by low-dose of IMI, although no significant histopathological changes were observed in the low-dose group. Six differential metabolites in the hippocampus and 10 differential metabolites in the liver were identified as the possible biomarkers to distinguish IMI exposure from the control group using the variable importance in projection (VIP) value and receiver operating characteristic (ROC) analysis. The metabolism disturbances of important biochemical pathways in the hippocampus and liver of mice in the exposed groups were elucidated, mostly concentrated in lipid metabolism, amino acid metabolism, nucleotide metabolism, carbohydrate metabolism, and energy metabolism (*p* < 0.05). Such investigations give out a global view of IMI-induced damages in the hippocampus and liver of mice and imply a health risk associated with early metabolic damage in mice.

## Introduction

Neonicotinoids are one new chemical class of insecticides developed and applied in the 1990s, following organophosphorus, carbamate and pyrethroid insecticides. Because of their systemic character and high efficiency to insect controls, neonicotinoids have become one of the fastest growing and the biggest selling insecticides in the market once introduced^1^. Neonicotinoids have been currently registered and approved for use on hundreds of field crops in more than 120 countries^2^. Up to August 2014, a total of 2076 products of 10 varieties of neonicotinoids had been registered in China, accounting for about 7% of the total number of pesticide registrations. The statistical data on July 30, 2017 showed that there were as many as 72 and 60 domestic registered manufacturers of imidacloprid (IMI) and thiamethoxam (TMX), respectively, and the registered contents of their original drugs were above 95%.

However, owing to the widespread application combined with the persistency and high leaching potential, neonicotinoids’ concentrations in waters, soils and foods have been found to exceed the residue limit standard in many countries, which greatly increase the exposure risk to non-target species worldwide^3,4^. Several studies have indicated that neonicotinoids are frequently detected in the general human populations of China^5,6^. On September 2018, the European Union (EU) issued new rules banning the sales and outdoor use of neonicotinoids^7^. It was surely mainly because of the high toxicity of neonicotinoids to bees. The potential toxicity to humans and their residues in the environment were also the reason why the EU made this decision. Neonicotinoids are considered to be insect-specific, as they can block the transmission function of central nervous system of insects by activating nicotinic acetylcholine receptors (nAChRs) on the postsynaptic membrane^8^. However, the evidence has been emerging that neonicotinoids have an adverse effect on mammalian *α*4*β*2 nAChR subtype^9^. Several experiments *in vivo* and *in vitro* suggested that exposure to neonicotinoids could induce neurotoxicity, hepatotoxicity, reproductive toxicity, and genotoxicity, which might be related to the affinity and distribution of the metabolites of neonicotinoids^10,11^.

IMI is one of the most frequently used neonicotinoid insecticides, which has been extensively applied in agricultural crop protection, the prevention and control of household pet pests and outdoor lawn pests. Some evidences have shown that IMI could cause neurotoxicity in mammals^12,13^. A prevalence case-control study in Japan indicated a significant association between neonicotinoids exposure and typical symptoms including neurological findings (OR = 14, 95% CI: 3.5–57)^14^. Animal experiments pointed out that exposure to IMI at over 45 mg/kg/day for 28 days significantly decreased the spontaneous locomotor activity including in horizontal and vertical movements and stimulated the pain sensation along with pathological changes in the brain of rats^12,13^. Liver is responsible for the metabolic detoxification of IMI and is also the main target organ that IMI damage. Toxicological experiments in rats demonstrated that exposure to IMI at 20 mg/kg/day for 60 days led to liver damage including central venous dilatation and congestion, hepatocyte degeneration, and elevated serum transaminase levels^15–17^. The hepatotoxicity test on mice showed that after exposure to IMI at 10 mg/kg/day for 28 days, the liver of mice showed obvious histopathological changes^18^; while exposure to IMI at 15 mg/kg/day for 15 days, the liver tissue of mice was damaged, and serum glutamate oxaloacetate transaminase, serum glutamate pyruvate kinase, alkaline phosphatase and total bilirubin were significantly increased^19^.

Most of the recent studies have focused on time-dose-response relationship of IMI-induced toxicity^15–20^. A smaller number of studies have sought to uncover the toxic mechanisms of IMI on mammals. Metabolomics was developed in the late 1990s and aims at the changes in small-molecule metabolite profiles caused by stressors in a biological cell, bio-fluids or tissue^21^ by using either nuclear magnetic resonance spectroscopy or mass spectrometry^22^. Metabolomics, acting as end points of gene expression and functional protein actions, provides a direct “functional readout of the physiological state” of an organism which facilitates to better understand the toxicant-induced responses and discover biomarkers^23,24^. Recently, a large number of literatures have described metabolomics studies in toxic effects induced by environmental pollutants, such as perfluorinated compounds^25,26^, pesticide residues^27,28^, heavy metals^29^, and nanoparticles^30^. Tufi *et al*.^28^ employed metabolomics to explore IMI-induced toxicity in the central nervous system of the freshwater snail and pointed out a disruption of neuronal metabolism. They found a turnover increase between choline and acetylcholine and proposed a hypothesis of an increase in the cholinergic gene expression. However, until now, no systematic metabolomics studies on mammals exposed to IMI have been reported.

Hence, in the present study, the histopathological, biochemical and metabolic alterations induced by repeated oral administration of two doses of IMI (5 and 20 mg/kg/day) for 28 days on hippocampus and liver of female KM mice were examined. A high-throughput untargeted metabolomics method was employed to evaluate effects of IMI on metabolic profiles in the hippocampus and liver of mice and identify the potential biomarkers for IMI exposure by ultrahigh performance liquid chromatography/hybrid quadrupole time-of-flight mass spectrometry (UPLC/Q-TOF MS).

## Results and Discussion

### Body weight and relative organ weight

Repeated oral administration of IMI at 5 and 20 mg/kg/day did not produce any signs of mortality. The initial and final body weight, net body weight gain and relative organ weight of brain and liver were given in Table 1. The results showed that there was no difference in initial body weight of mice among the control group, the low-dose group and the high-dose group. As listed in Table 1, there was a significant decrease in net body weight gain in the high-dose group of 20 mg/kg/day together with significant toxicity symptoms such as piloerection, tremor, diarrhea and salivation. However, when compared with the control group, significant increases in relative organ weight of brain and liver were observed in the high-dose group (20 mg/kg/day), implying that the toxic symptoms of organs had developed.

**Table 1:** Body weight and relative organ weight of mice.

Group	Initial body weight (g)	Final body weight (g)	Net body weight gain (g)	Relative organ weight (%)	
				Brain	Liver
Control	26.25 ± 0.31	33.75 ± 0.24	7.50 ± 0.39	1.14 ± 0.03	4.26 ± 0.10
Low	27.75 ± 0.58	35.40 ± 1.02	7.65 ± 0.48	1.21 ± 0.02	4.32 ± 0.12
High	27.73 ± 1.26	32.91 ± 0.88	5.18 ± 0.69^*^	1.39 ± 0.03^***^	4.70 ± 0.13^*^

Data presented as mean ± SE; N = 10 per group. Relative organ weight = organ weight/body weight × 100. *p < 0.05; ***p < 0.001.

### Behavior analysis

**Figure 1:**
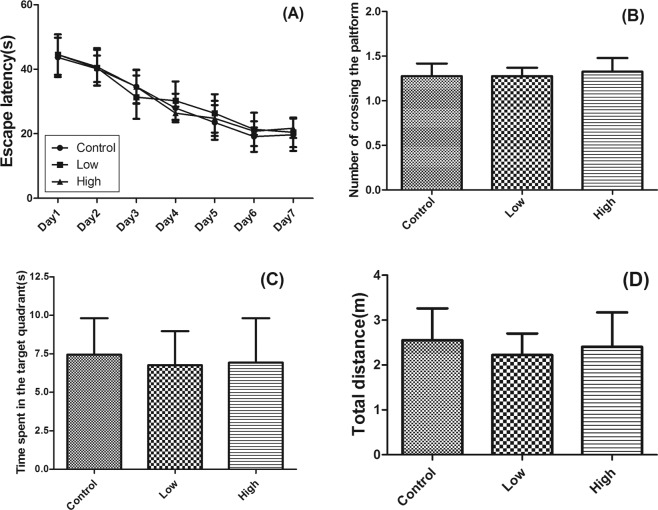
Effects of IMI treatment on behavior analysis of mice for 28 days by Morris water maze test. Data presented as mean ± SE; N = 10 per group. (**A**) Escape latency; (**B**) Number of crossing the platform; (**C**) Time spent in the target quadrant; (**D**) Total distance.

All mice underwent a Morris water maze to test their ability of learning and memory. The test process was divided into the six-days of space exploration training (Fig. 1A) and probe test on the seventh day (Fig. 1A–D). The results were analyzed and evaluated by recording the escape latency and total distance of mice. During the space exploration training, there was no significant difference in the escape latency among 3 groups (Fig. 1A). Additionally, there was no statistical difference in the escape latency (Fig. 1A), number of crossing the platform (Fig. 1B), time spent in the target quadrant (Fig. 1C), and total distance (Fig. 1D) in probe test among 3 groups, although the histopathological results (see section Histopathological examination) had shown that the hippocampus of mice had obvious visible damages. This may be due to that current histopathological injury was not sufficient to cause behavioral obstacle.

### Histopathological examination

**Figure 2:**
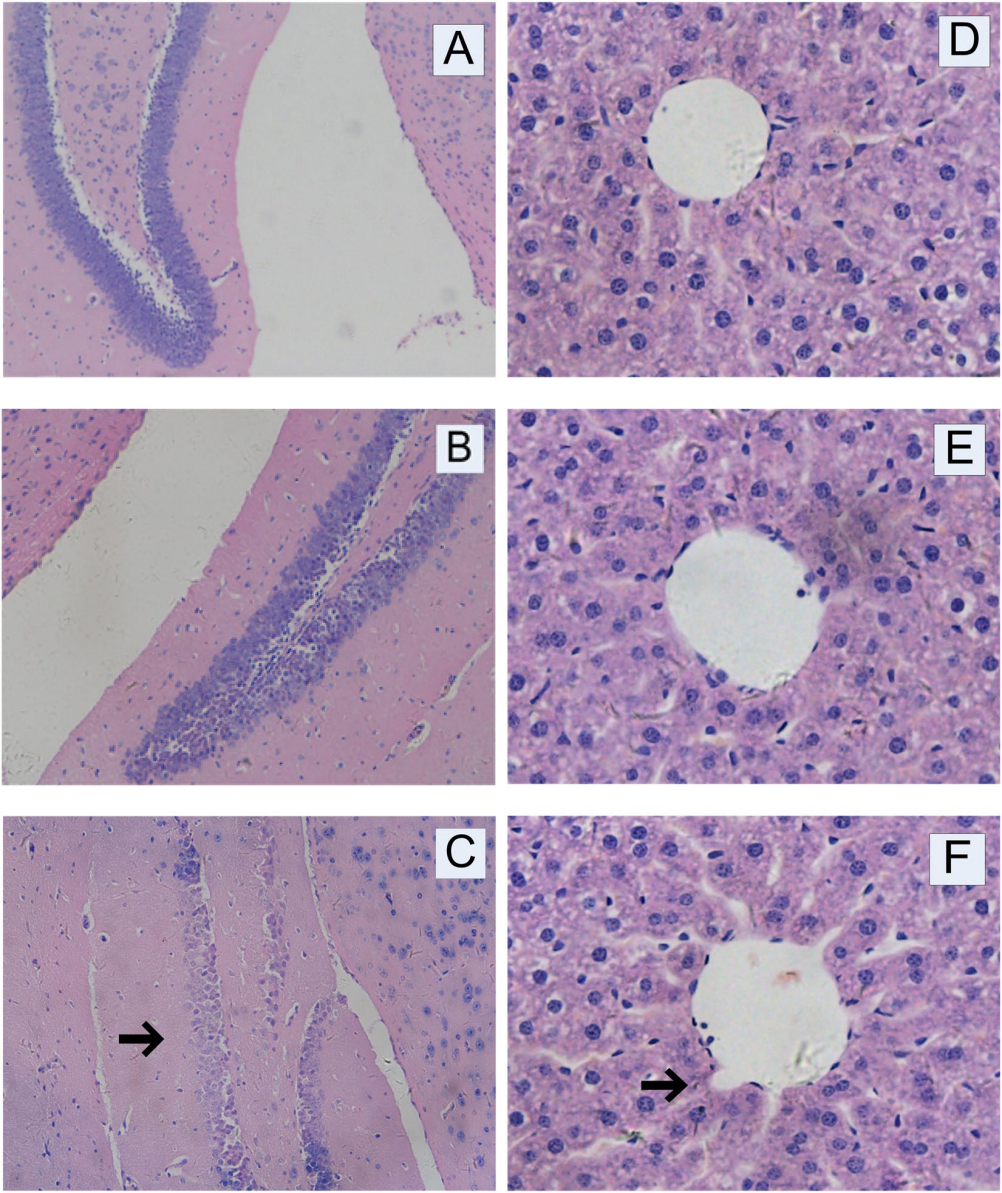
Effects of IMI treatment on pathological histology of hippocampus (A, Control; B, Low; C, High; 200×) and liver (D, Control; E, Low; F, High; 400×) of mice for 28 days.

The histopathological examinations of the hippocampus and liver of mice were carried out using hematoxylin-eosin staining. The results of the hippocampus were presented in Fig. 2A–C. Compared with the control group, there was no significant change in the low-dose group. However, the cell band of the hippocampal formation was significantly absent in the high-dose group, suggesting an IMI-induced toxicity in the brain of mice at the dose of 20 mg/kg/day. The histopathological examination results of the liver were presented in Fig. 2D–F. The results clearly showed that there was no significant change between the control group and the low-dose group. Compared with the control group, the liver appeared obvious vascular swelling in the high-dose group. The hepatic cords arranged around the central vein were disordered and liver cell necrosis and nuclear pyknosis were observed. The results above indicated that IMI produced significant subacute toxicity in the brain and liver of mice at 20 mg/kg/day which were in agreement with previous findings in Badgujar’s study^31^ and Arfat’s study^19^.

### Biochemical analysis

To investigate the effects of IMI exposure on the functional activity of liver enzymes, the plasma biochemical parameters of mice orally administered IMI including alanine aminotransferase (ALT), aspartate aminotransferase (AST) and alkaline phosphatase (AKP) were examined. The results were listed in Table 2. Compared with the control group, there was no significant change in the low-dose group. However, oral administration of IMI to mice at the high dose for 28 days resulted in significant increases of the plasma ALT, AST and AKP, implying the liver cell injury and degeneration.

**Table 2:** Biochemical parameters in plasma of mice.

Parameter	Control	Low	High
ALT (U/l)	27.58 ± 1.57	30.94 ± 1.65	37.13 ± 1.02^**^
AST (U/l)	97.25 ± 7.33	113.92 ± 8.47	135.52 ± 6.32^**^
AKP (U/l)	74.31 ± 5.34	82.37 ± 6.16	96.22 ± 2.51^**^

Data presented as mean ± SE; N = 6 per group. **p < 0.01.

### Quality control validation and UPLC/Q-TOF MS data processing

Pearson correlation analysis was performed on quality control (QC) samples to evaluate the system stability during UPLC/Q-TOF MS instrument analysis process and the results were presented in Supporting Information Fig. S1. As shown in Fig. S1, all the correlation coefficients (*r*) for the hippocampus and liver QC samples in positive and negative models were more than 0.9, indicating that the analysis system was stable and all the data were under control.

According to the UPLC/Q-TOF MS results, 6444 ion peaks for hippocampus and 11652 ion peaks for liver were detected and processed using XCMS program (https://xcmsonline.scripps.edu/landing_page.php?pgcontent=mainPage) for feature detection, retention time (t_R_) correction and alignment. The identification of the metabolites was operated in terms of accurate m/z value (<25 ppm) and MS/MS spectra by comparing with available reference standard mass spectral databases, Mass Bank (http://www.massbank.jp/) and Metlin library (http://metlin.scripps.edu/), and an in-house database (Shanghai Applied Protein Technology) established with authentic standards. In total, 116 endogenous metabolites from hippocampus and 50 endogenous metabolites from liver were identified which exhibited statistically significant differences (*p* < 0.05) among 3 groups. Heap maps produced by clustering of these differential metabolites were presented in Figs. S2 and S3. Among them, 47 metabolites from hippocampus and 31 metabolites from liver exhibited statistically significant differences (*p* < 0.05) between the control group and the exposed groups (Tables S1 and S2).

### Multivariate analysis of UPLC/Q-TOF MS data

**Figure 3:**
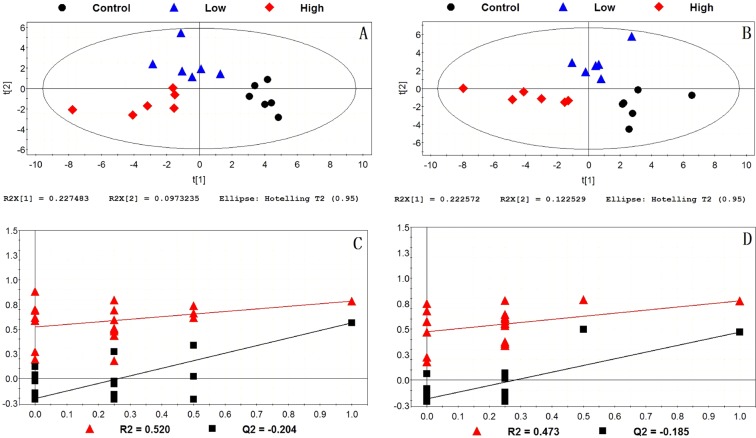
PLS-DA score plots and permutation tests of hippocampus among the control group, the low-dose group and the high-dose group. (**A**) PLS-DA score plot in positive model; (**B**) PLS-DA score plot in negative model; (**C**) validation of PLS-DA score plot in positive model; (**D**) validation of the PLS-DA score plot in negative model.

**Figure 4:**
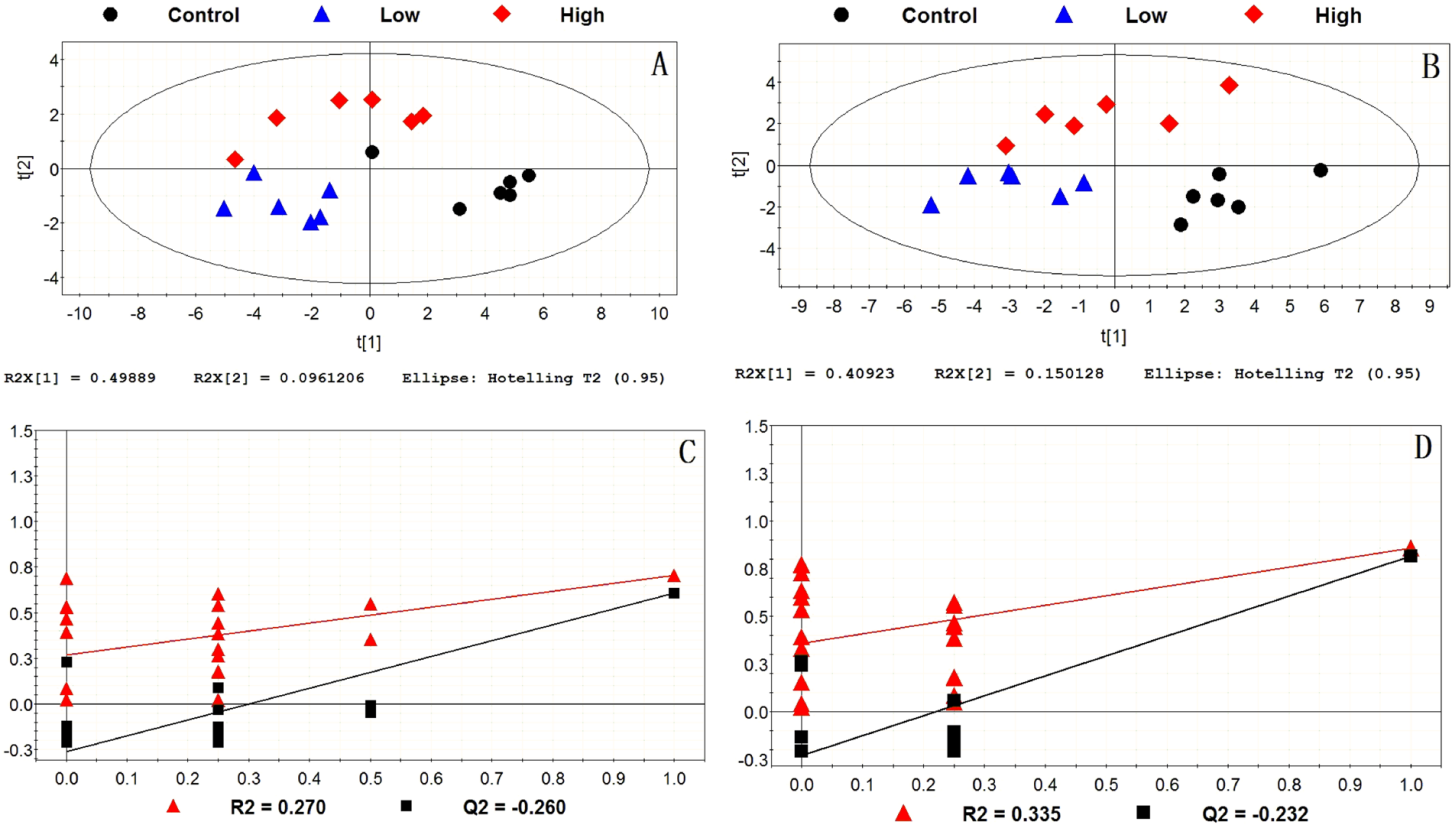
PLS-DA score plots and permutation tests of liver among the control group, the low-dose group and the high-dose group. (**A**) PLS-DA score plot in positive model; (**B**) PLS-DA score plot in negative model; (**C**) validation of PLS-DA score plot in positive model; (**D**) validation of the PLS-DA score plot in negative model.

The supervised partial least squares-discriminant analysis (PLS-DA) was used for modeling differences among 3 groups. The PLS-DA score plots and permutation tests for the hippocampus and liver samples in positive and negative models were shown in Figs. 3 and 4. It could be seen that all data points in the PLS-DA score plots were located within the Hotelling T2 (95%) ellipse. Permutation tests were performed to evaluate the fitting degree of PLS-DA after modeling the data. R^2^ stands for the explanation capacity of the model and Q^2^ stands for the predictive capacity of the model^32^. The low values of intercepts, R^2^ (<0.520) and Q^2^ (<−0.185), showed that the built models were not over-fitting (Figs. 3 and 4)). The correct rates of these models were more than 83.33%. The score plots presented a clear separation among 3 groups both in the hippocampus and liver, indicating that exposure to IMI induced metabolism disturbances in the hippocampus and liver of mice. Note that, compared with the control group, the metabolic profile in the low-dose group of 5 mg/kg/day had changed, although no significant histopathological changes were observed between the control group and the low-dose group (Fig. 2). This revealed a metabolism disruption and a health risk associated to early metabolic injury by exposure to low-dose of IMI.

### Potential biomarkers identification

The potential biomarkers for IMI exposure have been comprehensively analyzed and filtered by the variable importance in projection (VIP) value and receiver operating characteristic (ROC) analysis. The ROC curve was constructed by plotting the sensitivity against the corresponding false-positive rate (1-specificity). The differential metabolites with the area under curve (AUC) > 0.70 and with VIP value > 1.0 were selected as potential biomarkers. The sensitivity, specificity, and youden index for the selected cut-off of each parameter were given in Table 3.

**Table 3:** Potential biomarkers for IMI exposure discovered by VIP value and ROC analysis.

	Biomarkers	VIP	*p* value	AUC	Sensitivity (%)	Specificity (%)	Youden index
**Hippocampus**							
Control–Low	Acetylcholine	2.36	**	0.917	83.3	100.0	0.833
	sn-Glycerol 3-phosphoethanolamine	1.18	*	0.861	83.3	83.3	0.666
	L-Phenylalanine	1.43	*	0.778	83.3	66.7	0.500
Control–High	Acetylcholine	3.23	***	1.000	100.0	100.0	1.000
	D-Lyxose	1.31	**	0.944	83.3	100.0	0.833
	Adenine	1.22	*	0.806	66.7	100.0	0.667
	GMP	1.07	*	0.861	83.3	83.3	0.666
**Liver**							
Control–Low	L-Glutamine	2.92	***	1.000	100.0	100.0	1.000
	S-Lactoylglutathione	2.26	*	0.972	100.0	83.3	0.833
	Inosine	7.32	*	0.889	100.0	66.7	0.667
	AMP	3.53	*	0.889	100.0	66.7	0.667
	L-Pyroglutamic acid	1.50	*	0.806	100.0	66.7	0.667
	Adenine	1.25	*	0.889	83.3	83.3	0.666
	2-Hydroxyadenine	1.24	*	0.833	83.3	83.3	0.666
Control–High	Guanosine	2.03	*	0.889	100.0	66.7	0.667
	UDP-N-acetylglucosamine	1.92	*	0.806	100.0	66.7	0.667
	L-Pyroglutamic acid	1.73	*	0.861	100.0	66.7	0.667
	Adenine	1.57	*	0.889	100.0	66.7	0.667
	2-Hydroxyadenine	1.53	*	0.889	100.0	66.7	0.667
	1-Stearoyl-2-hydroxy-sn-glycero-3-phosphocholine	3.11	*	0.778	83.3	83.3	0.666

Abbreviations: AUC, area under curve; GMP, Guanosine 5′-monophosphate; AMP, Adenosine monophosphate. *p < 0.05; **p < 0.01; ***p < 0.001.

As described in Table 3, in the hippocampus, 3 metabolites including acetylcholine, sn-glycerol 3-phosphoethanolamine and L-phenylalanine were identified as the possible biomarkers to distinguish low-dose of IMI exposure from the control group. When the exposure dose of IMI increased, the metabolic profile changed. This led to significant changes in the concentration of 4 metabolites including acetylcholine, D-lyxose, adenine and GMP, which could be used as the potential biomarkers. It’s worth noting that with the increase of the exposure dose of IMI, the difference of acetylcholine level was more obvious. Compared with the low-dose group, the higher AUC (1.000) and youden index (1.000) of acetylcholine were obtained between the control group and the high-dose group. This revealed that acetylcholine as the potential biomarker could provide a satisfactory distinction of IMI exposure from the controls and a better prediction of early metabolic damage of an organism induced by IMI exposure. The orthogonal partial least squares-discriminant analysis (OPLS/O2PLS-DA) loading plots were employed to further identify the variables that strongly contribute to the separation of classes. The x axis, the loading p, denotes the influence of the variables and the y axis (po) denotes the credibility of the variables^33^. From Figs. S4 and S5, we could still detect these potential biomarkers concentrated in the lower left corner of the plots, which exhibited a reliability of identified biomarkers.

In the liver, 7 metabolites including L-glutamine, S-lactoylglutathione, inosine, AMP, L-pyroglutamic acid, adenine and 2-hydroxyadenine were identified as the possible biomarkers to distinguish low-dose of IMI exposure from the control group. Among them, L-glutamine was discovered with the highest AUC (1.000) and youden index (1.000) between the control group and the low-dose group. The excellent sensitivity and specificity of L-glutamine revealed that L-glutamine was expected to be a biomarker to predict early metabolic damage by exposure to low-dose of IMI. With the increase of the exposure dose of IMI, the metabolic profile changed. Six metabolites including guanosine, UDP-N- acetylglucosamine, L-pyroglutamic acid, adenine, 2-hydroxyadenine and 1-stearoyl-2- hydroxyl-sn-glycero-3-phosphocholine were identified as the possible biomarkers to distinguish high-dose IMI exposure from the control group. Among them, 3 metabolites including L-pyroglutamic acid, adenine and 2-hydroxyadenine were selected at two exposed concentrations at the same time, implying their good monitoring and early warning for IMI exposure. In addition, as shown in Figs. S6 and S7, these potential biomarkers were found to be furthest from the origin, exhibiting a reliability of identified biomarkers.

### Metabolic pathway and function analysis

**Figure 5:**
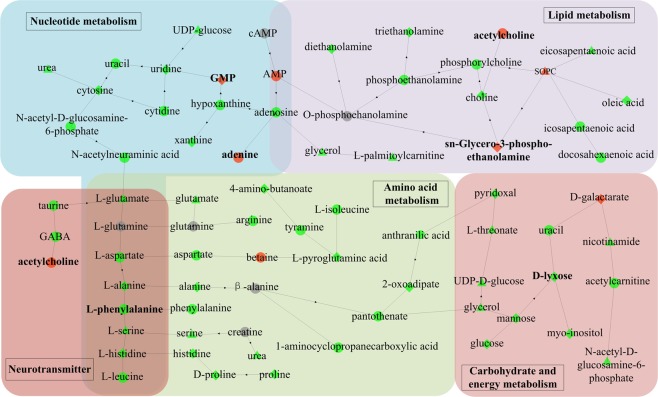
Disturbed biochemical pathways for the comparison between the control group and the exposed groups in the hippocampus. The color and shape of the nodes represent the fold changes between different groups: Red, up-regulation; Green, down-regulation; Gray, no statistical difference; Triangle, Control–Low; Diamond, Control–High; Dot, Control–Low and Control–High. The bold without borders represents the selected biomarkers.

**Figure 6:**
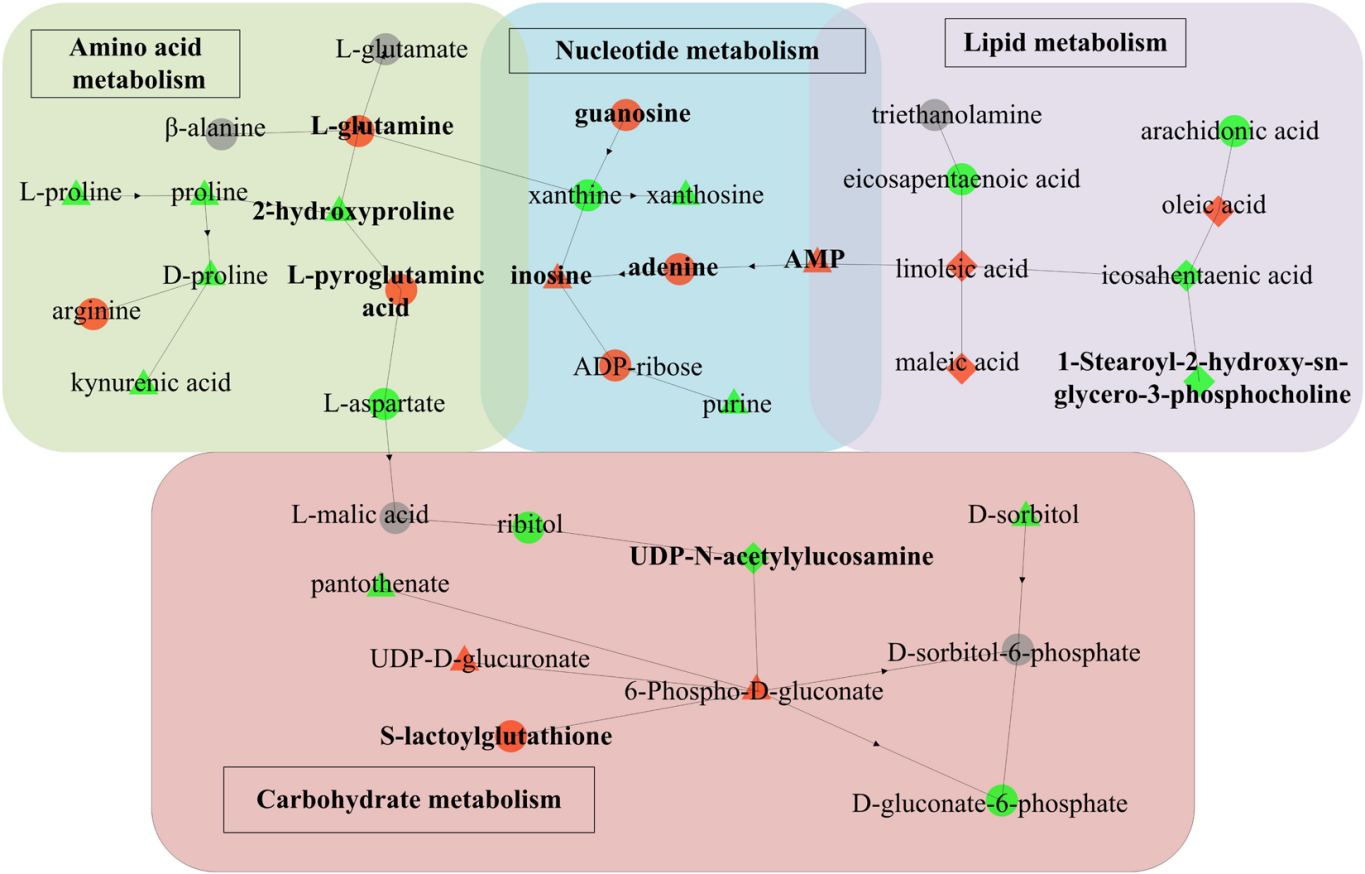
Disturbed biochemical pathways for the comparison between the control group and the exposed groups in the liver. The color and shape of the nodes represent the fold changes between different groups: Red, up-regulation; Green, down-regulation; Gray, no statistical difference; Triangle, Control–Low; Diamond, Control–High; Dot, Control–Low and Control–High. The bold without borders represents the selected biomarkers.

Based on the results of differential metabolites, we further investigated the relevant metabolic pathways affected by IMI in the hippocampus and liver. The Kyoto Encyclopedia of Genes and Genomes (KEGG) enrichment analysis^34–36^ and Fisher’s Exact Test were employed to analyze and calculate the significance level of differential metabolite enrichment in each pathway, so as to determine the metabolism pathways that were significantly affected. The pathway enrichment analysis results (Tables S1 and S2) showed that in the low-dose group, 27 differential metabolites in the hippocampus participated in 6 metabolic cycles including lipid metabolism, amino acid metabolism, nucleotide metabolism, carbohydrate metabolism, energy metabolism, and metabolism of cofactors and vitamins (*p* < 0.05), while 22 differential metabolites in the liver mainly participated in 4 metabolic cycles including amino acid metabolism, nucleotide metabolism, carbohydrate metabolism, and metabolism of other secondary metabolites (*p* < 0.05). The alteration in these metabolic pathways might be the initial response to IMI exposure, since histopathological changes had not been induced yet. The disruption of certain metabolic pathways might return to normal through compensatory responses. However, certain metabolic disorders might be exacerbated with the increase of exposure time, eventually leading to tissue damage in mice. It could be seen from Fig. 5, when exposed to high-dose of IMI, glycerophospholipid metabolism, 5 amino acid metabolism pathways, pyrimidine metabolism, and pantothenate and CoA biosynthesis were still affected, whereas the disturbance of biosynthesis of unsaturated fatty acids and purine metabolism were appeared in the hippocampus. In the liver (Fig. 6), purine metabolism was still affected by exposure to high-dose of IMI, whereas the disturbance of biosynthesis of unsaturated fatty acids, D-glutamine and D-glutamate metabolism, glyoxylate and dicarboxylate metabolism, and pyruvate metabolism were appeared.

The glycerophospholipid metabolism in the hippocampus of mice was found to be affected by exposure to IMI at two exposed concentrations. In this metabolic pathway, phosphocholine, choline, phosphoethanolamine, and triethanolamine were significantly downregulated (*p* < 0.05), whereas acetylcholine was significantly upregulated (*p* < 0.05) in the high-dose group. This suggested a disruption of cholinergic system relating with the mode of action of IMI^28^. Acetylcholine was identified as a potential biomarker for IMI exposure due to its excellent sensitivity and specificity. The up-regulation of acetylcholine might be due to that IMI bound competitively to the acetylcholine receptors (nAChRs) on the postsynaptic membrane of neuronal cells, leading to the accumulation of acetylcholine. The decrease of choline and the increase of acetylcholine implied the inhibition of acetylcholinesterase (AchE) and choline O-acetyltransferase (ChAT).

The dysregulation of biosynthesis of unsaturated fatty acids in the hippocampus and liver were appeared in the high-dose group. The levels of arachidonic acid and eicosapentaenoic acid were found to be decreased, whereas an increase in oleic acid and linoleic acid were found. Derivatives of long-chain polyunsaturated fatty acids such as arachidonic acid and eicosapentaenoic acid were important mediators of inflammation^37^. Inflammatory mediators derived from eicosapentaenoic acid such as resolvins and protectin had anti-inflammatory effects^38^. Arachidonic acid was mainly used to synthesize pro-inflammatory mediators. After binding to inflammatory cell receptors, arachidonic acid activated intracellular inflammatory signal transduction and promoted the synthesis and release of inflammatory factors such as tumor necrosis factor and interleukin, leading to amplification of the inflammatory response^39^.

An alteration in many amino acids in the hippocampus and liver were observed, involving arginine biosynthesis, alanine, aspartate and glutamate metabolism, D-glutamine and D-glutamate metabolism, glycine, serine and threonine metabolism, valine, and leucine and isoleucine biosynthesis. The metabolism including amino acids was known as an important part of neurotransmitter processing^40^. The levels of 9 amino acid neurotransmitters involving L-aspartate, L-glutamate, L-glutamine, L-isoleucine, L-leucine, taurine, L-serine, L- phenylalanine and *γ*-aminobutyric acid (GABA) were found to be significantly downregulated (*p* < 0.05). Especially, L-phenylalanine could be used as a potential biomarker to assess the metabolic disturbance in the hippocampus caused by exposure to low-dose of IMI.

Additionally, the disturbance of amino acid metabolism could affect the gut-associated immune system^41–43^ and initiate gut inflammation^44–46^ via the modulation of key metabolic signaling pathways. Changes in some amino acids such as L-glutamine, L-glutamate, L-aspartate, L-leucine and L-serine could effectively impact bacterial composition, diversity and activity, and modulate macrophages and dendritic cells^47^. Meanwhile, L-glutamine could regulate the compartmental amino acid metabolism via a variety of pathways, thus further affecting the amino acid composition and content in the gut^48^.

The metabolism of other amino acids was also dysregulated by exposure to IMI involving *β*-alanine metabolism, taurine and hypotaurine metabolism, and the glutathione metabolism. The glutathione metabolism was usually correlated to oxidative stress^49^. Glutathione was involved in the process of cell membrane protection and free radical scavenging. It could help cells maintain normal immune system functions, and had antioxidant and integrative detoxification effects. The alteration of glutathione metabolism suggested that IMI could trigger the overproduction of free radicals leading to oxidative stress in the hippocampus and liver of mice, which was in agreement with previous findings in Duzguner’s studies^50,51^. The levels of L-pyroglutamic acid, S-lactoylglutathione and L-glutamine associated with oxidative stress were found to be increased, which could be used as the potential biomarkers for IMI exposure.

In addition, nucleotides were involved as well and, as a consequence, purine metabolism and pyrimidine metabolism were dysregulated by exposure to IMI. In the hippocampus, the levels of adenine and GMP were found to be increased, whereas the levels of adenosine, hypoxanthine and xanthine were found to be decreased in the high-dose group. In the liver, the levels of adenine, guanosine and ADP-ribose were found to be increased, whereas the level of xanthine was found to be decreased in the high-dose group. Among them, 3 differential metabolites involving adenine, GMP and guanosine were identified as the potential biomarkers for IMI exposure due to their good sensitivity and specificity.

From the metabolomics results, there was no significant difference in hormones between the control group and the exposed groups, and the disturbance of hormone-related pathways were not appeared by GEGG pathway enrichment analysis. These exhibited the intergroup consistency of hormone levels and indicated that repeated oral administration of IMI at two different doses did not cause changes in hormone levels, which were in agreement with previous findings in Vohra’s study^16^ and Kapoor’s study^52^.

## Conclusions

In the present study, the histopathological, biochemical and metabolic alterations induced by oral administration of two doses of IMI (5 and 20 mg/kg/day) for 28 days on hippocampus and liver of mice were studied. Compared with the control group, the hippocampus and liver of mice appeared significant histopathological changes in the high-dose group (20 mg/kg/day). Although no obvious damages were observed in the low-dose group, multivariate statistical analysis presented 3 different metabolic profiles for the control group, the low-dose group and the high-dose group. The altered metabolic profile in the low-dose group (5 mg/kg/day) indicated that the metabolism disturbance in the hippocampus and liver of mice had been induced by low-dose of IMI, implying a health risk associated with early metabolic damage in mice. Six major metabolic cycles in the hippocampus and 4 major metabolic cycles in the liver were affected by exposure to IMI. Six metabolites in the hippocampus and 10 metabolites in the liver were discovered as the possible biomarkers for IMI exposure. The neurotransmitter acetylcholine in the hippocampus and L-glutamine and S-lactoylglutathione associated with oxidative stress in the liver as the potential biomarkers (AUC > 0.9; youden index > 0.8) could provide a satisfactory prediction of early metabolic damage of an organism induced by IMI exposure. Such investigations give out a global view of IMI-induced damages in the hippocampus and liver of mice. Our results may facilitate further research aimed at understanding the toxic mechanisms of IMI on mammals and developing the early intervention of IMI exposure combined with useful biomarkers.

## Materials and Methods

### Chemicals and reagents

Technical grade IMI (>98% purity), MS-grade ammonium acetate, and MS-grade ammonium hydroxide were purchased from Sigma-Aldrich (Schnelldorf, Germany). HPLC-grade acetonitrile and methanol were purchased from Merck (Darmstadt, Germany). Milli-Q water was obtained from a Millipore purification system (Millipore, Milford, MA, USA).

### Animals and diet

Animal experiments were approved by the Ethics Committee of Chongqing Medical University (Chongqing, China), and all experiments were carried out in accordance with the approved guidelines. Female KM mice (6-8-week-old), individual-ventilated-cages grade, were obtained from Chongqing Medical University Laboratory Animal Center. Female mice were chosen because female mice were reported more sensitive to the toxic effects of neonicotinoids^18,53,54^ and have been widely used in metabolomics studies of the toxic effects induced by environmental pollutants^55–58^. The mice were housed in a climate-controlled room with a temperature of 20–25 °C, a humidity of 30–60%, and a 12 h light/dark cycle. The mice were given standard diet containing pelleted food and water ad libitum. All mice were acclimatized for one week before using them for experiments.

### Experimental design and sample collection

After acclimatization for one week, the mice were randomly divided into 3 groups. One group was served as control and was given corn oil as vehicle through oral intubation. The other two groups were given 5 and 20 mg/kg/day (corresponding to 1/30th and 1/7.5th LD_50_ of IMI) IMI suspended in corn oil to mice for 28 days, respectively. Body weights were recorded throughout the period of experiments. After 28-day exposure, the mice of each group were used in behavioral study. Then, blood samples were collected from the retro-orbital sinus in heparin anticoagulation tube. Finally, all the mice were sacrificed by cervical dislocation. The mice used in the metabolomics analysis were in the stage of estrus by visual observation of the external genitalia (the vaginal opening was swollen, pink and moist) described by Champlin’s group^59^. The brain and liver were quickly harvested and weighted. The hippocampus and liver samples were rinsed with phosphate-buffered saline (PBS) and stored at −80 °C until analysis.

### Morris water maze test

A circular stainless steel poor was divided into 4 quadrants marking with square, triangle, circle and tree, respectively. Flour was used to turn the water white and the water temperature was kept at 23 ± 2 °C. A 9-cm-diameter escape platform was positioned in one of the quadrants submerged 0.5-1 cm above the surface of water. Screened mice had vision problems or did not swim were excluded for following experiments. The mice were trained 4 times a day at 4 different starting positions facing the pool wall with 20-min intervals for 6 days. In each trial, mice were given 60 s to find the platform. If the mice could not find the platform within the specified time, they were placed directly on the platform for 15 s. The swim speed, distances (path length), and time (escape latency) to the platform were monitored and calculated with a video camera linked to a computer system using MWM software (WMT-100S Morris water maze, Techman, Sichuan, China). On day 7, a probe test was performed to evaluate spatial memory ability. The platform was removed from the pool and the mice were placed in a new starting position 180° from the original platform position to swim freely in the poor for 60 s. The number of crossings over the original position of the platform and the time spent in the target quadrant were recorded.

### Histopathological examination

The hippocampus and liver samples were cleared from adhering tissues, placed in 4% paraformaldehyde fix solution. After routine processing, paraffin section of each tissue was cut at 5 μm thicknesses and stained with hematoxylin and eosin for light microscopic examination.

### Biochemical analysis

Plasma was separated by centrifuging the blood at 3000 rpm for 15 min at 4 °C and frozen immediately at −20 °C until analysis. The plasma ALT, AST and AKP were examined using standard kits which were purchased from Beyotime (Shanghai, China).

### Analysis of metabolic profiles

After all the samples were slowly thawed at 4 °C, 60 mg of each tissue sample was added with 200 μL of pre-cooled Milli-Q water, and homogenized. Then 800 μL of pre-cooled methanol/acetonitrile (1:1, v/v) was added and vortexed. i) The hippocampal tissue was sonicated in ultrasonic bath for 30 min at low temperature, stood for 10 min at −20 °C, and centrifuged at 14000 rpm for 20 min at 4 °C. The supernatants were dried in vacuum. The residues were reconstituted in 100 μL of acetonitrile-water (1:1, v/v), centrifuged at 14000 rpm for 15 min at 4 °C. The resulting supernatants were taken for UPLC/Q-TOF MS analysis. ii) The liver tissue was sonicated in ultrasonic bath twice at low temperature (each time for 30 min), incubated for 1 h at −20 °C to precipitate proteins, and centrifuged at 13000 rpm for 15 min at 4 °C. The supernatants were taken for UPLC/Q-TOF MS analysis.

Analyses were performed using an UPLC system (1290 Infinity LC, Agilent Technologies) coupled to a Triple TOF 5600 system (AB Sciex, USA) with electrospray ionization (ESI) in the positive and negative modes. The ESI source conditions were set as follows: ion source gas (GS1 and GS2), 60 psi; curtain gas (CUR), 30 arbitrary units; source temperature, 600 °C; ion spray voltage floating (ISVF), ±5500 V. In MS only acquisition, the instrument was set to acquire in the m/z range of 60-1000 Da, and the accumulation time for TOF MS scan was set at 0.20 s/spectra. In auto MS/MS acquisition, the instrument was set to acquire in the m/z range of 25-1000 Da, and the accumulation time for product ion scan was set at 0.05 s/spectra. The product ion scan was acquired using information dependent acquisition (IDA) with high sensitivity mode. The collision energy (CE) was 35 ± 15 eV. Declustering potential (DP) was ±60 V. For chromatographic separation, an ACQUIY UPLC BEH Amide column (2.1 mm × 100 mm, 1.7 µm; waters, Ireland) was used, maintaining at 25 °C. The mobile phase contained water with 25 mM ammonium acetate and 25 mM ammonium hydroxide (A) and acetonitrile (B). A gradient elution program was used wherein the ratio of A:B varied as follows: 0 min, 5% A; 1.0 min, 5% A; 14.0 min, 35% A; 16.0 min, 60% A; 18.0 min, 60% A; 18.1 min, 5% A; 23.0 min, 5% A. The gradients were at a flow rate of 0.3 mL/min. A 2 µL aliquot of each sample was injected.

### Statistical analysis

Statistical analyses were performed using SPSS 22.0 software (IBM Corporation, Armonk, NY, USA). After the determination of the normality of the data distribution, one-way “analysis of variance (ANOVA)” with *post-hoc* Fisher’s least significant difference (LSD) test was applied to assess statistical differences among multiple groups. A *p* < 0.05 was considered to be statistically significant. The results were reported as the mean value ± standard error (SE) and displayed using GraphPad Prism version 5.0 statistical software (GraphPad, Inc., La Jolla, USA).

Partial least squares-discriminant analysis (PLS-DA) was carried out using SIMCA-P 14.1 (Umetrics, Umea, Sweden) to model differences among the control group and two exposed groups. The potential biomarkers for IMI exposure were comprehensively analyzed and filtered by variable importance in projection (VIP) value, receiver operating characteristic (ROC) analysis and orthogonal partial least squares-discriminant analysis (OPLS/O2PLS-DA) loading plots. Hierarchical clustering analysis was performed using the open-source cross-platform software Cluster 3.0 (http://bonsai.hgc.jp/~mdehoon/software/cluster/software. htm) coupled with Java Treeview package (http://www.java.com/). The altered metabolism pathways were analyzed using the Kyoto Encyclopedia of Genes and Genomes (KEGG) pathway enrichment analysis. The disturbed biochemical pathways and key metabolites were displayed by Microsoft Office Visio 2007 (Microsoft Corporation, Redmond, WA, USA).

## Supplementary information

Supplementary information.

## References

[1] Bass C, Denholm I, Williamson MS, Nauen R (2015). The global status of insect resistance to neonicotinoid insecticides. Pestic. Biochem. Physiol..

[2] Jeschk P, Nauen R, Schindler M, Elbert A (2011). Overview of the status and global strategy for neonicotinoids. J. Agric. Food Chem..

[3] Morrissey CA (2015). Neonicotinoid contamination of global surface waters and associated risk to aquatic invertebrates: A review. Environ. Int..

[4] Goulson D (2013). Review: an overview of the environmental risks posed by neonicotinoid insecticides. J. Appl. Ecol..

[5] Wang L (2015). Occurrence and profile characteristics of the pesticide imidacloprid, preservative parabens, and their metabolites in human urine from rural and urban China. Environ. Sci. Technol..

[6] Zhang Q (2018). Simultaneous determination of nine neonicotinoids in human urine using isotope-dilution ultra-performance liquid chromatography-tandem mass spectrometry. Environ. Pollut..

[7] Butler, D. Scientists hail European ban on bee-harming pesticides. *Nature News*, 10.1038/d41586-018-04987-4 (2018).

[8] Tomizawa M, Casida JE (2005). Neonicotinoid insecticide toxicology: mechanisms of selective action. Annu. Rev. Pharmacol. Toxicol..

[9] Li P, Ann J, Akk G (2011). Activation and modulation of human α4β2 nicotinic acetylcholine receptors by the neonicotinoids clothianidin and imidacloprid. J. Neurosci. Res..

[10] Han W, Tian Y, Shen X (2018). Human exposure to neonicotinoid insecticides and the evaluation of their potential toxicity: An overview. Chemosphere.

[11] Wang X (2018). Mechanism of neonicotinoid toxicity: Impact on oxidative stress and metabolism. Annu. Rev. Pharmacol. Toxicol..

[12] Lonare M (2014). Evaluation of imidacloprid-induced neurotoxicity in male rats: a protective effect of curcumin. Neurochem. Int..

[13] Kimura-Kuroda J, Komuta Y, Kuroda Y, Hayashi M, Kawano H (2012). Nicotine like effects of the neonicotinoid insecticides acetamiprid and imidacloprid on cerebellar neurons from neonatal rats. PLoS One.

[14] Marfo JT (2015). Relationship between urinary N-desmethyl-acetamiprid and typical symptoms including neurological findings: A prevalence case-control study. PLoS One.

[15] Bhardwaj S, Srivastava MK, Kapoor U, Srivastava LP (2010). A 90 days oral toxicity of imidacloprid in female rats: morphological, biochemical and histopathological evaluations. Food Chem. Toxicol..

[16] Vohra P, Khera KS, Sangha GK (2014). Physiological, biochemical and histological alterations induced by administration of imidacloprid in female albino rats. Pestic. Biochem. Physiol..

[17] Kapoor U, Srivastava MK, Trivedi P, Garg V, Srivastava LP (2014). Disposition and acute toxicity of imidacloprid in female rats after single exposure. Food Chem. Toxicol..

[18] Badgujar PC (2013). Immunotoxic effects of imidacloprid following 28 days of oral exposure in BALB/c mice. Environ. Toxicol. Pharmacol..

[19] Arfat Y (2014). Effect of imidacloprid on hepatotoxicity and nephrotoxicity in male albino mice. Toxicol. Rep.

[20] Toor HK, Sangha GK, Khera KS (2013). Imidacloprid induced histological and biochemical alterations in liver of female albino rats. Pestic. Biochem. Physiol..

[21] Nicholson JK, Lindon JC (2008). Systems biology: metabonomics. Nature.

[22] Blow N (2008). Biochemistry’s new look. Nature.

[23] Nicholson JK, Lindon JC, Holmes E (1999). ‘Metabonomics’: understanding the metabolic responses of living systems to pathophysiological stimuli via multivariate statistical analysis of biological NMR spectroscopic data. Xenobiotica.

[24] Nicholson JK, Connelly J, Lindon JC, Holmes E (2002). Metabonomics: a platform for studying drug toxicity and gene function. Nat. Rev. Drug. Discov..

[25] Yu N (2016). Effects of perfluorooctanoic acid on metabolic profiles in brain and liver of mouse revealed by a high-throughput targeted metabolomics approach. Sci. Rep..

[26] Wang L (2014). PFOS induced lipid metabolism disturbances in BALB/c mice through inhibition of low density lipoproteins excretion. Sci. Rep.

[27] Teng M (2018). Metabolomics and transcriptomics reveal the toxicity of difenoconazole to the early life stages of zebrafish (Danio rerio). Aquat. Toxicol..

[28] Tufi S, Stel JM, de Boer J, Lamoree MH, Leonards PE (2015). Metabolomics to explore imidacloprid-induced toxicity in the central nervous system of the freshwater snail lymnaea stagnalis. Environ. Sci. Technol..

[29] Huang Q (2016). Integrated proteomics and metabolomics analysis of rat testis: Mechanism of arsenic-induced male reproductive toxicity. Sci. Rep..

[30] Wu B, Zhu L, Le XC (2017). Metabolomics analysis of TiO_2_ nanoparticles induced toxicological effects on rice (Oryza sativa L.). Environ. Pollut..

[31] Badgujar PC (2013). Immunotoxic effects of imidacloprid following 28 days of oral exposure in BALB/c mice. Environ. Toxicol. Pharmacol..

[32] Umetrics, A. User Guide to SIMCA-P+. Version 12, USA, Umetrics Inc. (2008).

[33] Wiklund S (2008). Visualization of GC/TOF-MS-based metabolomics data for identification of biochemically interesting compounds using OPLS class models. Anal. Chem..

[34] Kanehisa M, Goto S (2000). KEGG: Kyoto encyclopedia of genes and genomes. Nucleic Acids Res..

[35] Kanehisa M, Sato Y, Furumichi M, Morishima K, Tanabe M (2019). New approach for understanding genome variations in KEGG. Nucleic Acids Res..

[36] Kanehisa M (2019). Toward understanding the origin and evolution of cellular organisms. Protein Sci..

[37] Dhingra AK, Chopra B, Dass R, Mittal SK (2015). An update on anti-inflammatory compounds: a review. Antiinflamm. Antiallergy. Agents Med. Chem..

[38] Yates CM, Calder PC, Ed Rainger G (2014). Pharmacology and therapeutics of omega-3 polyunsaturated fatty acids in chronic inflammatory disease. Pharmacol. Ther..

[39] Trostchansky A, Rubbo H (2017). Anti-inflammatory signaling actions of electrophilic nitro-arachidonic acid in vascular cells and astrocytes. Arch. Biochem. Biophys..

[40] Pasantes-Morales H, Franco R, Ochoa L, Ordaz B (2002). Osmosensitive release of neurotransmitter amino acids: relevance and mechanisms. Neurochem. Res..

[41] Zackular JP, Skaar EP (2018). The role of zinc and nutritional immunity in Clostridium difficile infection. Gut microbes..

[42] Elkrief A, Derosa L, Zitvogel L, Kroemer G, Routy B (2019). The intimate relationship between gut microbiota and cancer immunotherapy. Gut microbes..

[43] Sterlin D (2019). Immune/microbial interface perturbation in human IgA deficiency. Gut microbes..

[44] Kiely CJ, Pavli P, O’Brien CL (2018). The role of inflammation in temporal shifts in the inflammatory bowel disease mucosal microbiome. Gut microbes..

[45] Miki T, Okada N, Hardt WD (2018). Inflammatory bactericidal lectin RegIIIβ: Friend or foe for the host?. Gut microbes..

[46] Sokol H (2018). Specificities of the intestinal microbiota in patients with inflammatory bowel disease and Clostridium difficile infection. Gut microbes..

[47] He L (2018). Autophagy: the last defense against cellular nutritional stress. Adv. Nutr..

[48] Ma N, Ma X (2019). Dietary amino acids and the gut-microbiome-immune axis: physiological metabolism and therapeutic prospects. Compr. Rev. Food Sci. Food Saf..

[49] Diaz-Vivancos P, de Simone A, Kiddle G, Foyer CH (2015). Glutathione-linking cell proliferation to oxidative stress. Free. Radic. Biol. Med..

[50] Duzguner V, Erdogan S (2012). Chronic exposure to imidacloprid induces inflammation and oxidative stress in the liver & central nervous system of rats. Pestic. Biochem. Physiol..

[51] Duzguner V, Erdogan S (2010). Acute oxidant and inflammatory effects of imidacloprid on the mammalian central nervous system and liver in rats. Pestic. Biochem. Physiol..

[52] Kapoor U, Srivastava MK, Srivastava LP (2011). Toxicological impact of technical imidacloprid on ovarian morphology, hormones and antioxidant enzymes in female rats. Food Chem. Toxicol..

[53] Terayama H (2018). Effect of acetamiprid on the immature murine testes. Int. J. Environ. Health Res..

[54] Kataria SK, Chhillar AK, Kumar A, Tomar M, Malik V (2016). Cytogenetic and hematological alterations induced by acute oral exposure of imidacloprid in female mice. Drug. Chem. Toxicol..

[55] Du X (2020). Metabolomics analysis of urine from healthy wild type mice exposed to ambient PM_2.5_. Sci. Total. Environ..

[56] Zhao C (2019). Serum metabolomics analysis of mice that received repeated airway exposure to a water-soluble PM2.5 extract. Ecotoxicol. Environ. Saf..

[57] LeGouëllec A, Moyne O, Meynet E, Toussaint B, Fauvelle F (2018). High-resolution magic angle spinning NMR-based metabolomics revealing metabolic changes in lung of mice infected with P. aeruginosa consistent with the degree of disease severity. J. Proteome Res..

[58] Tu LN (2017). Metabolomic characteristics of cholesterol-induced non-obese nonalcoholic fatty liver disease in mice. Sci. Rep..

[59] Champlin AK, Dorr DL, Gates AH (1973). Determining the stage of the estrous cycle in the mouse by the appearance of the vagina. Biol. Reprod..

