# Supplementary material for: Metabolic disturbance in hippocampus and liver of mice: A primary response to imidacloprid exposure
Source: Sci Rep. 2020 Mar 31;10:5713. doi: 10.1038/s41598-020-62739-9 (PMC7109098; doi:10.1038/s41598-020-62739-9)
Supplement: Supplementary file 1 — Supplementary information. [file 41598_2020_62739_MOESM1_ESM.doc]

Supplementary Materials

**Metabolic disturbance in hippocampus and liver of mice: A primary response to imidacloprid exposure**

Meilin Zheng ^a,1^, Qizhong Qin ^b,1^, Wenli Zhou ^a^, Qin Liu ^a^, Shaohua Zeng ^c^,

Hong Xiao ^a^, Qunhua Bai ^a^, Jieying Gao ^a,*^

^a^ School of Public Health and Management,

Chongqing Medical University, Chongqing 400016, P. R. China

^b^ Center of Experimental Teaching for Public Health,

Experimental Teaching and Management Center,

Chongqing Medical University, Chongqing 401331, P. R. China

^c^ China Coal Technology & Engineering Group Chongqing Research Institute,

Chongqing 400039, P. R. China

CONTENT

Table S1 Metabolite profiles in the hippocampus of mice. [1](#__RefHeading___Toc33562718)

Table S2 Metabolite profiles in the liver of mice. [8](#__RefHeading___Toc33562719)

Fig. S1. Correlation spectrum of QC samples. (A) in positive model; (B) in negative mode. [12](#__RefHeading___Toc33562720)

Fig. S2. Heap maps produced by clustering of the differential metabolites in the hippocampus using Cluster 3.0 software (http://bonsai.hgc.jp/~mdehoon/software/ cluster/software.htm) coupled with Java Treeview package (http://www.java.com/). (A) in positive model; (B) in negative mode. [13](#__RefHeading___Toc33562721)

Fig. S3. Heap maps produced by clustering of the differential metabolites in the liver using Cluster 3.0 software (http://bonsai.hgc.jp/~mdehoon/software/cluster/software. htm) coupled with Java Treeview package (http://www.java.com/). [15](#__RefHeading___Toc33562722)

(A) in positive model; (B) in negative mode. [15](#__RefHeading___Toc33562723)

Fig. S4. OPLS/O2PLS-DA loading plots in the hippocampus between the control group and the low-dose group. (A) in positive model; (B) in negative mode. [16](#__RefHeading___Toc33562724)

Fig. S5. OPLS/O2PLS-DA loading plots in the hippocampus between the control group and the high-dose group. (A) in positive model; (B) in negative mode. [17](#__RefHeading___Toc33562725)

Fig. S6. OPLS/O2PLS-DA loading plots in the liver between the control group and the low-dose group. (A) in positive model; (B) in negative mode. [18](#__RefHeading___Toc33562726)

Fig. S7. OPLS/O2PLS-DA loading plots in the liver between the control group and the high-dose group. (A) in positive model; (B) in negative mode. [19](#__RefHeading___Toc33562727)

# Table S1 Metabolite profiles in the hippocampus of mice.

| No. | Metabolites | t_R_  (min) | m/z | Control - Low | | |  | Control - High | | | KEGG  Pathway ^c^ | KEGG Compound |
| --- | --- | --- | --- | --- | --- | --- | --- | --- | --- | --- | --- | --- |
|  |  |  |  | VIP | *p* value | Trend ^a^ |  | VIP | *p* value | Trend ^b^ |  |  |
| ESI^+^ |  |  |  |  |  |  |  |  |  |  |  |  |
| 1 | (3-Carboxypropyl)  trimethylammonium cation | 11.83 | 146.1167 | 1.36 | * | ↓ |  | 1.63 | * | ↓ | - | - |
| 2 | (4Z,7Z,10Z,13Z,16Z,19Z)-4,7,10,13,1 6,19- Docosahexaenoic acid | 1.17 | 346.2734 | 1.76 | * | ↓ |  | 1.63 | * | ↓ | Biosynthesis of unsaturated fatty acids | C06429 |
| 3 | 1,2-dioleoyl-sn-glycero-  3-phosphatidylcholine | 4.32 | 786.6005 | 1.89 | * | ↓ |  |  |  |  | - | - |
| 4 | 2-Ethoxyethanol | 1.84 | 151.0954 | 2.13 | ** | ↓ |  | 2.14 | ** | ↓ | - | C14687 |
| 5 | Acetylcarnitine | 9.47 | 204.1228 | 5.16 | ** | ↓ |  | 5.77 | ** | ↓ | - | C02571 |
| 6 | Acetylcholine | 5.75 | 146.1166 | 2.36 | ** | ↑ |  | 3.23 | *** | ↑ | Glycerophospholipid metabolism;  cAMP signaling pathway;  Neuroactive ligand-receptor interaction;  Synaptic vesicle cycle; Cholinergic synapse;  Taste transduction;  Nicotine addiction | C01996 |
| 7 | Adenosine | 2.42 | 250.0930 | 1.19 | * | ↓ |  | 2.28 | *** | ↓ | Purine metabolism;  cGMP-PKG signaling pathway; cAMP signaling pathway; Sphingolipid signaling pathway; Neuroactive ligand-receptor interaction;  Regulation of lipolysis in adipocytes;  Renin secretion;  Parkinson's disease;  Morphine addiction;  Alcoholism | C00212 |
| 8 | Cyclohexylamine | 6.51 | 100.1112 | 2.13 | ** | ↓ |  | 1.46 | * | ↓ | - | - |
| 9 | Cytidine | 7.76 | 244.0919 | 1.29 | * | ↓ |  | 1.41 | *** | ↓ | Pyrimidine metabolism | C00475 |
| 10 | Diethanolamine | 6.17 | 88.0748 | 2.16 | ** | ↓ |  |  |  |  | Glycerophospholipid metabolism | C06772 |
| 11 | Eicosapentaenoic acid | 1.29 | 303.2319 | 2.24 | ** | ↓ |  |  |  |  | Biosynthesis of unsaturated fatty acids | C06428 |
| 12 | L-Aspartate | 13.14 | 134.0442 | 1.39 | * | ↓ |  | 1.23 | * | ↓ | Arginine biosynthesis;  Alanine, aspartate and glutamate metabolism;  Glycine, serine and threonine metabolism;  *β*-alanine metabolism;  Nicotinate and nicotinamide metabolism  Pantothenate and CoA biosynthesis;  Aminoacyl-tRNA biosynthesis;  ABC transporters;  Neuroactive ligand-receptor interaction;  Protein digestion and absorption;  Central carbon metabolism in cancer | C00049 |
| 13 | L-Glutamate | 12.58 | 148.0599 | 2.40 | * | ↓ |  |  |  |  | Arginine biosynthesis;  Alanine, aspartate and glutamate metabolism;  Taurine and hypotaurine metabolism;  D-glutamine and D-glutamate metabolism;  Glyoxylate and dicarboxylate metabolism;  Nitrogen metabolism;  Aminoacyl-tRNA biosynthesis  ABC transporters;  FoxO signaling pathway;  Neuroactive ligand-receptor interaction;  Synaptic vesicle cycle;  Retrograde endocannabinoid signaling;  Glutamatergic synapse;  GABAergic synapse;  Taste transduction;  Proximal tubule bicarbonate reclamation;  Protein digestion and absorption;  Huntington's disease;  Nicotine addiction;  Alcoholism;  Central carbon metabolism in cancer | C00025 |
| 14 | L-Isoleucine | 8.41 | 132.1007 | 1.45 | ** | ↓ |  | 1.85 | * | ↓ | Valine, leucine and isoleucine biosynthesis;  Aminoacyl-tRNA biosynthesis  ABC transporters;  Protein digestion and absorption;  Mineral absorption;  Central carbon metabolism in cancer | C00407 |
| 15 | L-Leucine | 8.05 | 132.1013 | 1.59 | ** | ↓ |  | 2.47 | ** | ↓ | Valine, leucine and isoleucine biosynthesis;  Aminoacyl-tRNA biosynthesis;  ABC transporters;  mTOR signaling pathway;  Protein digestion and absorption;  Mineral absorption;  Central carbon metabolism in cancer | C00123 |
| 16 | L-Palmitoylcarnitine | 4.79 | 400.3415 | 2.94 | * | ↓ |  |  |  |  | - | C02990 |
| 17 | L-Phenylalanine | 8.41 | 166.0852 | 1.43 | * | ↑ |  | 1.70 | ** | ↓ | Aminoacyl-tRNA biosynthesis;  ABC transporters;  Protein digestion and absorption;  Mineral absorption;  Central carbon metabolism in cancer | C00079 |
| 18 | Nicotinamide | 1.44 | 123.0544 | 1.82 | ** | ↓ |  |  |  |  | Nicotinate and nicotinamide metabolism; | C00153 |
| 19 | Phosphorylcholine | 15.43 | 184.0731 | 2.29 | * | ↓ |  | 5.87 | ** | ↓ | Glycerophospholipid metabolism;  Choline metabolism in cancer | C00588 |
| 20 | Taurine | 9.11 | 126.0210 | 3.64 | ** | ↓ |  | 1.75 | * | ↓ | Taurine and hypotaurine metabolism;  Sulfur metabolism;  ABC transporters;  Neuroactive ligand-receptor interaction | C00245 |
| 21 | Urea | 2.91 | 61.0389 | 1.08 | ** | ↓ |  |  |  |  | Arginine biosynthesis;  Purine metabolism;  Pyrimidine metabolism;  ABC transporters | C00086 |
| 22 | 1-Palmitoyl-sn-glycero-3-phosphocholine | 5.93 | 496.3398 |  |  |  |  | 3.64 | * | ↓ | - | - |
| 23 | Cytosine | 7.33 | 112.0496 |  |  |  |  | 1.35 | *** | ↓ | Pyrimidine metabolism | C00380 |
| 24 | D-Proline | 9.58 | 116.0697 |  |  |  |  | 1.06 | ** | ↓ | - | C00763 |
| 25 | L-Alanine | 10.88 | 134.0178 |  |  |  |  | 1.36 | ** | ↓ | Alanine, aspartate and glutamate metabolism;  Taurine and hypotaurine metabolism;  Aminoacyl-tRNA biosynthesis;  ABC transporters;  Protein digestion and absorption;  Mineral absorption;  Central carbon metabolism in cancer | C00041 |
| 26 | L-Histidine | 12.17 | 156.0759 |  |  |  |  | 1.30 | ** | ↓ | *β*-alanine metabolism;  Aminoacyl-tRNA biosynthesis;  ABC transporters;  Protein digestion and absorption;  Central carbon metabolism in cancer; | C00135 |
| 27 | L-Pyroglutamic acid | 9.33 | 130.0490 |  |  |  |  | 1.04 | * | ↓ | - | C01879 |
| 28 | Triethanolamine | 4.50 | 150.1114 |  |  |  |  | 1.16 | * | ↓ | Glycerophospholipid metabolism | C06771 |
| 29 | Tyramine | 7.85 | 120.0800 |  |  |  |  | 1.58 | ** | ↓ | Neuroactive ligand-receptor interaction;  Protein digestion and absorption | C00483 |
| 30 | Uridine | 4.81 | 489.1454 |  |  |  |  | 1.16 | * | ↓ | Pyrimidine metabolism | C00299 |
| 31 | Xanthine | 6.50 | 153.0400 |  |  |  |  | 1.97 | *** | ↓ | Purine metabolism | C00385 |
| ESI^- d^ |  |  |  |  |  |  |  |  |  |  |  |  |
| 1 | Glycerol | 3.21 | 151.0614 | 1.08 | ** | ↓ |  |  |  |  | ABC transporters  Retrograde endocannabinoid signaling;  Regulation of lipolysis in adipocytes | C00116 |
| 2 | L-Serine | 11.88 | 104.0354 | 1.12 | *** | ↓ |  |  |  |  | Glycine, serine and threonine metabolism;  Sphingolipid metabolism;  Glyoxylate and dicarboxylate metabolism;  Sulfur metabolism;  Aminoacyl-tRNA biosynthesis;  ABC transporters;  Sphingolipid signaling pathway;  Protein digestion and absorption  Mineral absorption;  Central carbon metabolism in cancer; | C00065 |
| 3 | L-Threonate | 10.50 | 135.0303 | 2.29 | * | ↓ |  |  |  |  | - | C01620 |
| 4 | N-Acetylneuraminic acid | 11.92 | 308.0992 | 1.41 | * | ↓ |  | 1.25 | ** | ↓ | - | C00357 |
| 5 | O-phosphoethanolamine | 14.88 | 140.0124 | 1.92 | * | ↓ |  | 1.84 | * | ↓ | Glycerophospholipid metabolism;  Sphingolipid metabolism;  Sphingolipid signaling pathway | C00346 |
| 6 | sn-Glycerol 3-phosphoethanolamine | 12.50 | 214.0489 | 1.18 | * | ↑ |  |  |  |  | Glycerophospholipid metabolism;  Ether lipid metabolism | C01233 |
| 7 | 2-Oxoadipic acid | 11.11 | 141.0174 |  |  |  |  | 5.22 | * | ↓ | - | C00322 |
| 8 | 4-Aminobutyric acid | 11.79 | 102.0562 |  |  |  |  | 2.30 | ** | ↓ | Alanine, aspartate and glutamate metabolism;  *β*-alanine metabolism;  Nicotinate and nicotinamide metabolism;  cAMP signaling pathway;  Neuroactive ligand-receptor interaction;  Synaptic vesicle cycle;  Retrograde endocannabinoid signaling;  GABAergic synapse;  Taste transduction;  Morphine addiction;  Nicotine addiction | C00334 |
| 9 | Adenine | 4.93 | 134.0472 |  |  |  |  | 1.22 | * | ↑ | - | - |
| 10 | DL-lactate | 7.29 | 89.0249 |  |  |  |  | 4.00 | * | ↓ | - | C00256/C00186 |
| 11 | D-Lyxose | 1.90 | 209.0667 |  |  |  |  | 1.31 | ** | ↑ | - | - |
| 12 | GMP | 14.74 | 362.0509 |  |  |  |  | 1.07 | * | ↑ | Purine metabolism;  cGMP-PKG signaling pathway;  Taste transduction | C00144 |
| 13 | L-Proline | 9.79 | 114.0562 |  |  |  |  | 1.35 | ** | ↓ | Aminoacyl-tRNA biosynthesis;  ABC transporters;  Protein digestion and absorption;  Mineral absorption;  Central carbon metabolism in cancer | C00148 |
| 14 | Oleic acid | 1.81 | 281.2488 |  |  |  |  | 4.48 | * | ↓ | Biosynthesis of unsaturated fatty acids | C00712 |
| 15 | Oxypurinol | 6.72 | 303.0550 |  |  |  |  | 1.26 | ** | ↓ | - | C07599 |
| 16 | Uracil | 2.27 | 111.0201 |  |  |  |  | 3.26 | * | ↓ | Pyrimidine metabolism;  *β*-alanine metabolism;  Pantothenate and CoA biosynthesis | C00106 |

Abbreviations: GMP, Guanosine 5'-monophosphate. **p* < 0.05; ***p* < 0.01; ****p* < 0.001

^a^ Arrow show increase or decrease in the low-dose group compared with the control group.

^b^ Arrow show increase or decrease in the high-dose group compared with the control group.

^c^ Enriched KEGG pathways with *p* < 0.05.

^d^ The duplicate metabolites in ESI^+^ are excluded.

# Table S2 Metabolite profiles in the liver of mice.

| No. | Metabolites | t_R_  (min) | m/z | Control - Low | | |  | Control - High | | | KEGG  Pathway ^c^ | KEGG Compound |
| --- | --- | --- | --- | --- | --- | --- | --- | --- | --- | --- | --- | --- |
|  |  |  |  | VIP | *p* value | Trend ^a^ |  | VIP | *p* value | Trend ^b^ |  |  |
| ESI^+^ |  |  |  |  |  |  |  |  |  |  |  |  |
| 1 | L-Glutamine | 11.65 | 147.0764 | 2.92 | *** | ↑ |  |  |  |  | Purine metabolism;  D-Glutamine and D-glutamate metabolism;  Glyoxylate and dicarboxylate metabolism;  Glutamatergic synapse;  GABAergic synapse;  Proximal tubule bicarbonate reclamation;  Protein digestion and absorption;  Mineral absorption;  Central carbon metabolism in cancer | C00064 |
| 2 | 1-Stearoyl-sn-glycerol | 7.21 | 341.3048 | 1.01 | ** | ↓ |  | 1.09 | ** | ↓ | - | - |
| 3 | Eicosapentaenoic acid | 1.36 | 303.2310 | 2.48 | ** | ↓ |  |  |  |  | Biosynthesis of unsaturated fatty acids | C06428 |
| 4 | 20-Hydroxyarachidonic acid | 1.26 | 362.2685 | 1.51 | * | ↓ |  |  |  |  | - | - |
| 5 | Purine | 3.35 | 121.0506 | 1.11 | * | ↓ |  |  |  |  | - | C15587 |
| 6 | D-Proline | 9.32 | 116.0705 | 2.03 | * | ↓ |  |  |  |  | Arginine and proline metabolism | C00763 |
| 7 | Adenine | 5.04 | 136.0616 | 1.25 | * | ↑ |  |  |  |  | Purine metabolism | C00147 |
| 8 | S-Lactoylglutathione | 12.10 | 380.1110 | 2.26 | * | ↑ |  |  |  |  | Pyruvate metabolism | C03451 |
| 9 | AMP | 13.67 | 348.0698 | 3.53 | * | ↑ |  |  |  |  | Purine metabolism;  FoxO signaling pathway;  mTOR signaling pathway;  PI3K-Akt signaling pathway;  Regulation of lipolysis in adipocytes;  Aldosterone synthesis and secretion | C00020 |
| 10 | 2-Hydroxyadenine | 7.84 | 152.0562 | 1.24 | * | ↑ |  | 1.53 | * | ↑ | - | - |
| 11 | L-Pyroglutamic acid | 11.96 | 130.0497 | 1.50 | * | ↑ |  | 1.73 | * | ↑ | - | C01879 |
| 12 | Hydroxyproline | 7.83 | 114.0547 | 1.18 | * | ↓ |  |  |  |  | Arginine and proline metabolism | C01157 |
| 13 | Guanosine | 7.84 | 284.0987 |  |  |  |  | 2.03 | * | ↑ | Purine metabolism | C00387 |
| 14 | Adenine | 5.04 | 136.0616 |  |  |  |  | 1.57 | * | ↑ | Purine metabolism | C00147 |
| 15 | 1-Stearoyl-2-hydroxy-sn-  glycero-3-phosphocholine | 6.29 | 524.3693 |  |  |  |  | 3.11 | * | ↑ | - | - |
| 16 | UDP-N-acetylglucosamine | 13.46 | 608.0869 |  |  |  |  | 1.92 | * | ↓ | - | C00043 |
| ESI^- d^ |  |  |  |  |  |  |  |  |  |  |  |  |
| 1 | Arachidonic Acid | 1.17 | 303.2317 | 12.84 | ** | ↓ |  |  |  |  | Biosynthesis of unsaturated fatty acids;  GnRH signaling pathway;  Regulation of lipolysis in adipocytes;  Aldosterone synthesis and secretion;  Leishmaniasis | C00219 |
| 2 | Xanthosine | 6.46 | 283.0675 | 2.57 | ** | ↓ |  |  |  |  | Purine metabolism;  Caffeine metabolism | C01762 |
| 3 | 6-Phospho-D-gluconate | 15.47 | 275.0168 | 1.40 | ** | ↓ |  | 1.35 | *** | ↓ | Pentose phosphate pathway | C00345 |
| 4 | Ribitol | 7.03 | 151.0627 | 1.80 | ** | ↓ |  |  |  |  | - | C00474 |
| 5 | Inosine | 6.44 | 267.0743 | 7.32 | * | ↑ |  |  |  |  | Purine metabolism | C00294 |
| 6 | (+-)12-HETE | 1.35 | 319.2262 | 2.86 | * | ↓ |  |  |  |  | - | - |
| 7 | D-Sorbitol | 8.99 | 181.0726 | 1.23 | * | ↓ |  |  |  |  |  | C00794 |
| 8 | Xanthine | 6.23 | 151.0284 | 5.29 | * | ↓ |  | 3.86 | * | ↓ | Purine metabolism;  Caffeine metabolism | C00385 |
| 9 | Kynurenic acid | 5.32 | 188.0361 | 1.01 | * | ↓ |  |  |  |  | - | C01717 |
| 10 | Pantothenate | 8.12 | 218.1044 | 1.83 | * | ↓ |  |  |  |  | - | C00864 |
| 11 | PGF2a | 5.02 | 353.2311 |  |  |  |  | 1.12 | ** | ↓ | - | - |
| 12 | Maleic acid | 12.47 | 115.0065 |  |  |  |  | 1.16 | * | ↓ | - | C01384 |
| 13 | Sunitinib | 0.75 | 397.2015 |  |  |  |  | 1.01 | * | ↑ | - | - |
| 14 | Linoleic acid | 1.23 | 279.2327 |  |  |  |  | 14.59 | * | ↑ | Biosynthesis of unsaturated fatty acids | C01595 |
| 15 | L-Malic acid | 12.47 | 133.0168 |  |  |  |  | 3.13 | * | ↓ | Glyoxylate and dicarboxylate metabolism;  Proximal tubule bicarbonate reclamation;  Central carbon metabolism in cancer | C00149 |

Abbreviations: AMP, Adenosine monophosphate. **p* < 0.05; ***p* < 0.01; ****p* < 0.001

^a^ Arrow show increase or decrease in the low-dose group compared with the control group.

^b^ Arrow show increase or decrease in the high-dose group compared with the control group.

^c^ Enriched KEGG pathways with *p* < 0.05.

^d^ The duplicate metabolites in ESI^+^ are excluded.


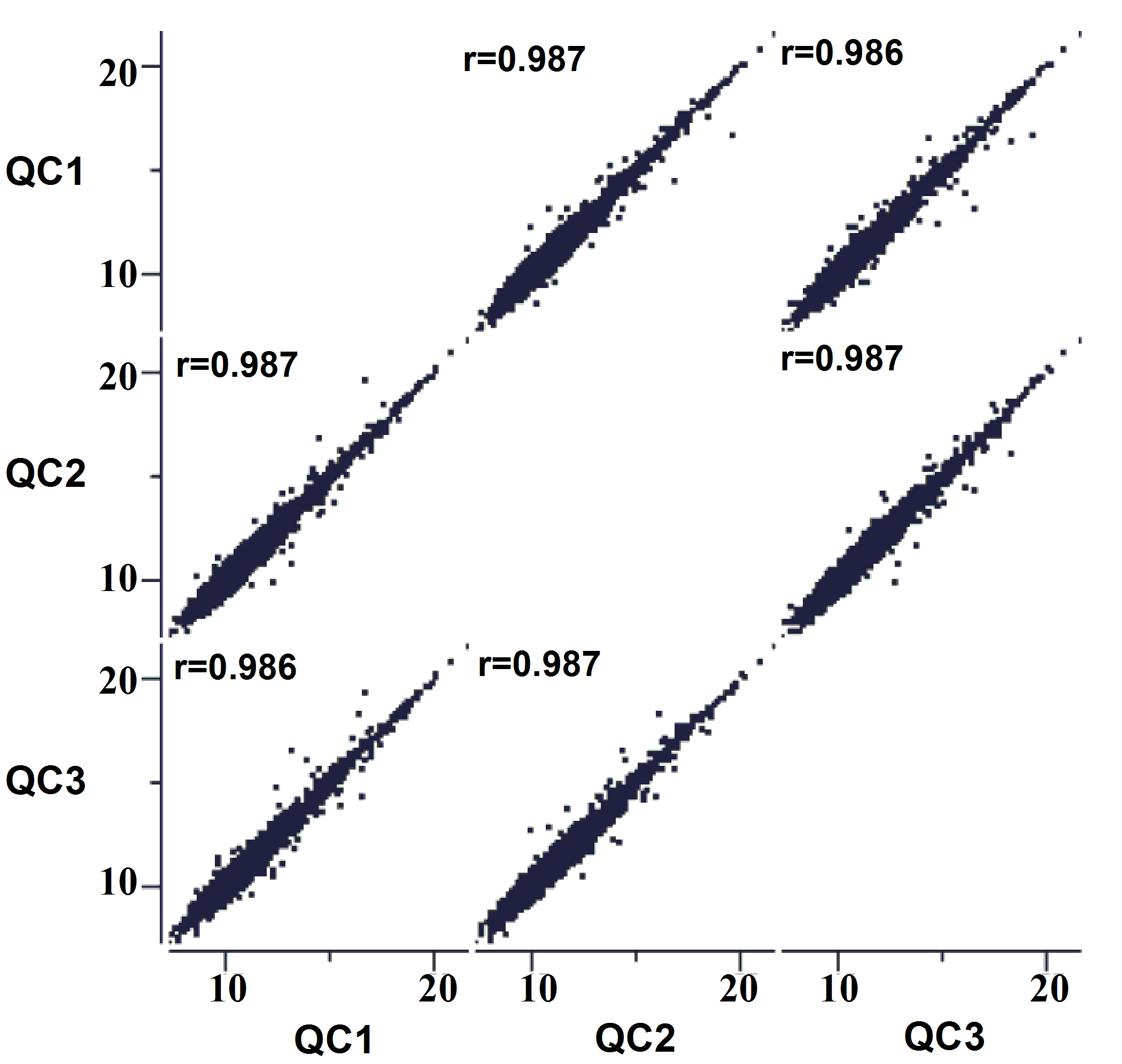


**(A)**


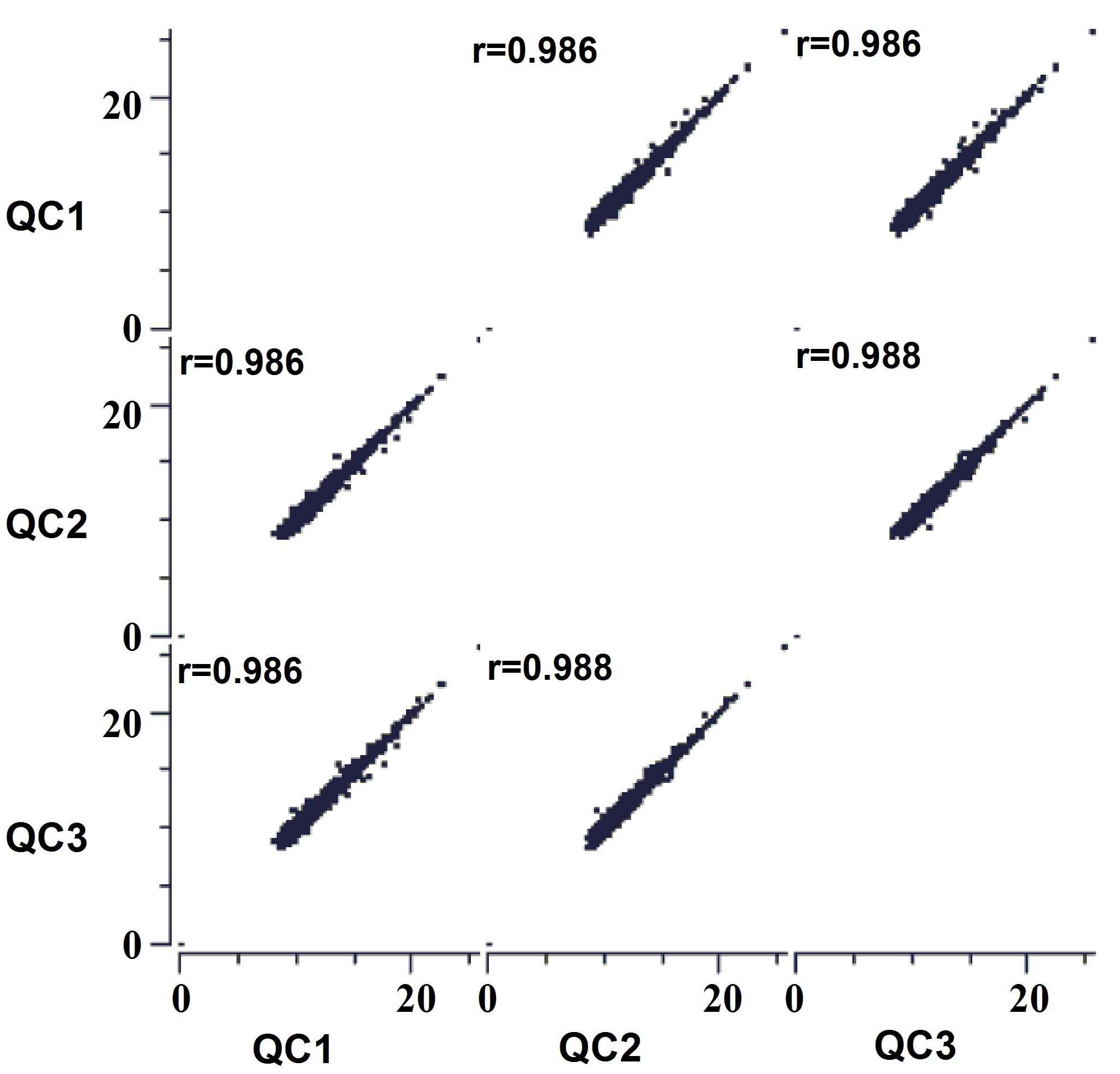


**(B)**

# Fig. S1. Correlation spectrum of QC samples. (A) in positive model; (B) in negative mode.


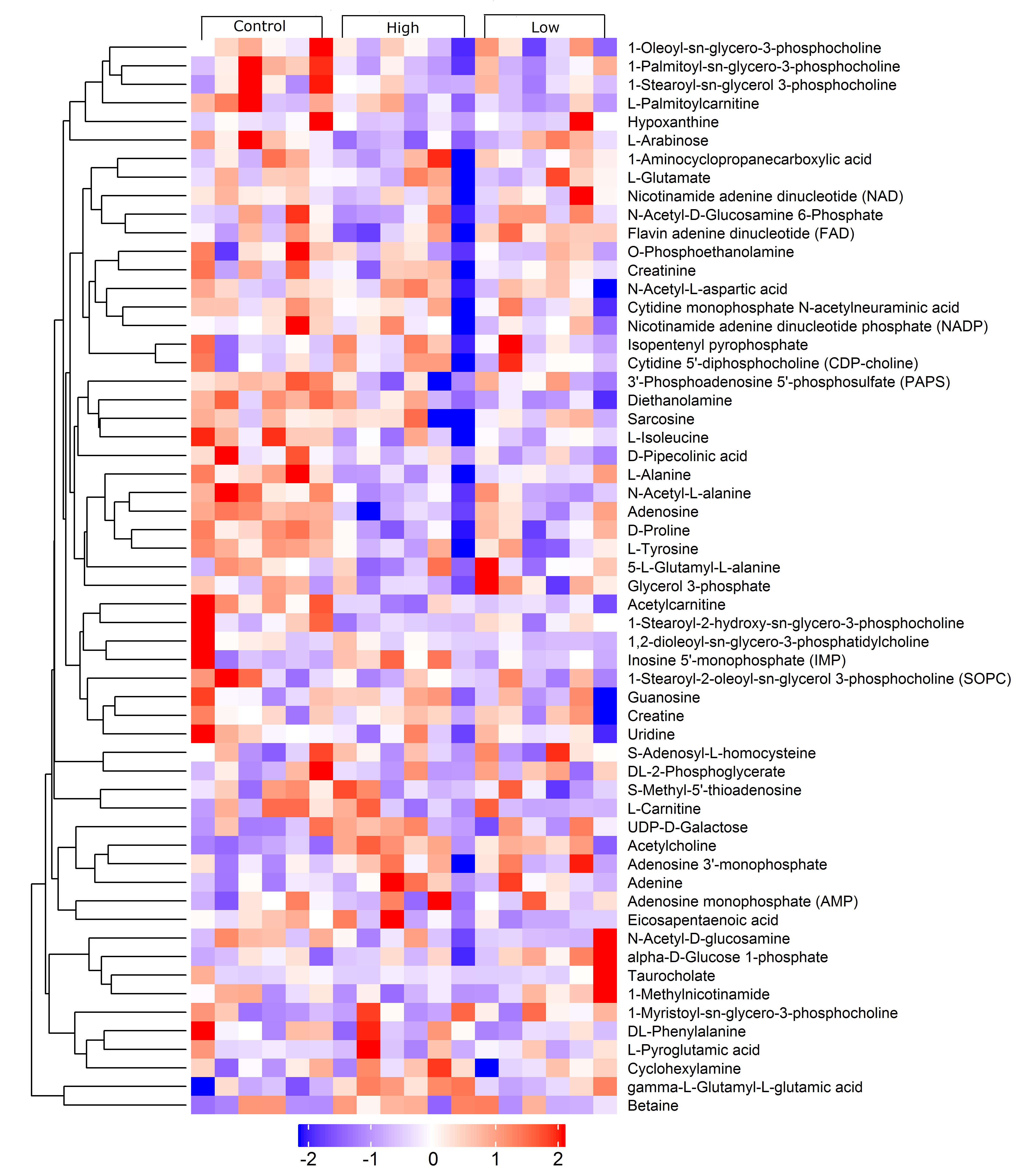


**(A)**

# Fig. S2. Heap maps produced by clustering of the differential metabolites in the hippocampus using Cluster 3.0 software ([**http://bonsai.hgc.jp/~mdehoon/software/**](http://bonsai.hgc.jp/~mdehoon/software/) cluster/software.htm) coupled with Java Treeview package (http://www.java.com/). (A) in positive model; (B) in negative mode.


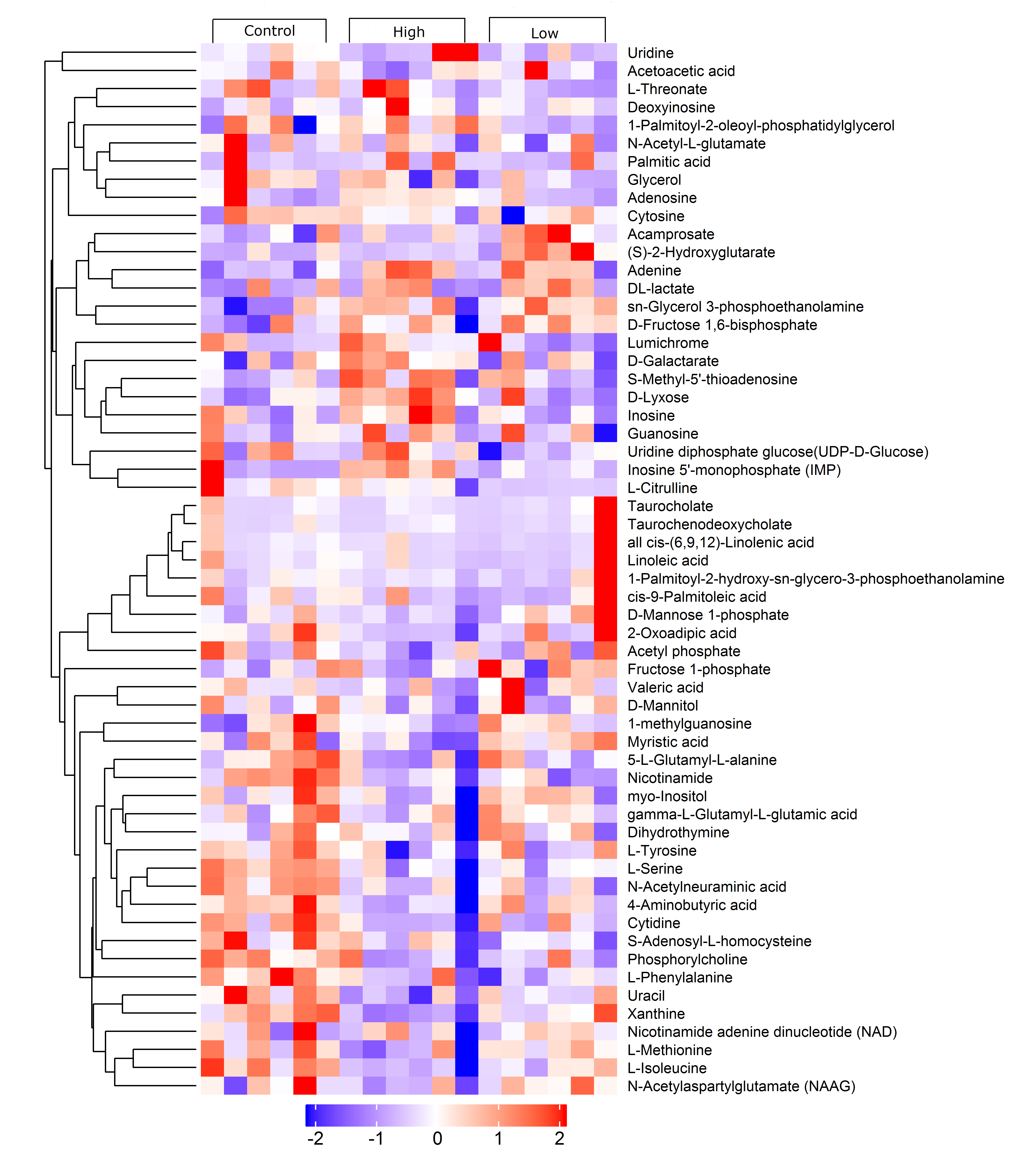


**(B)**

Fig. S2. Continued


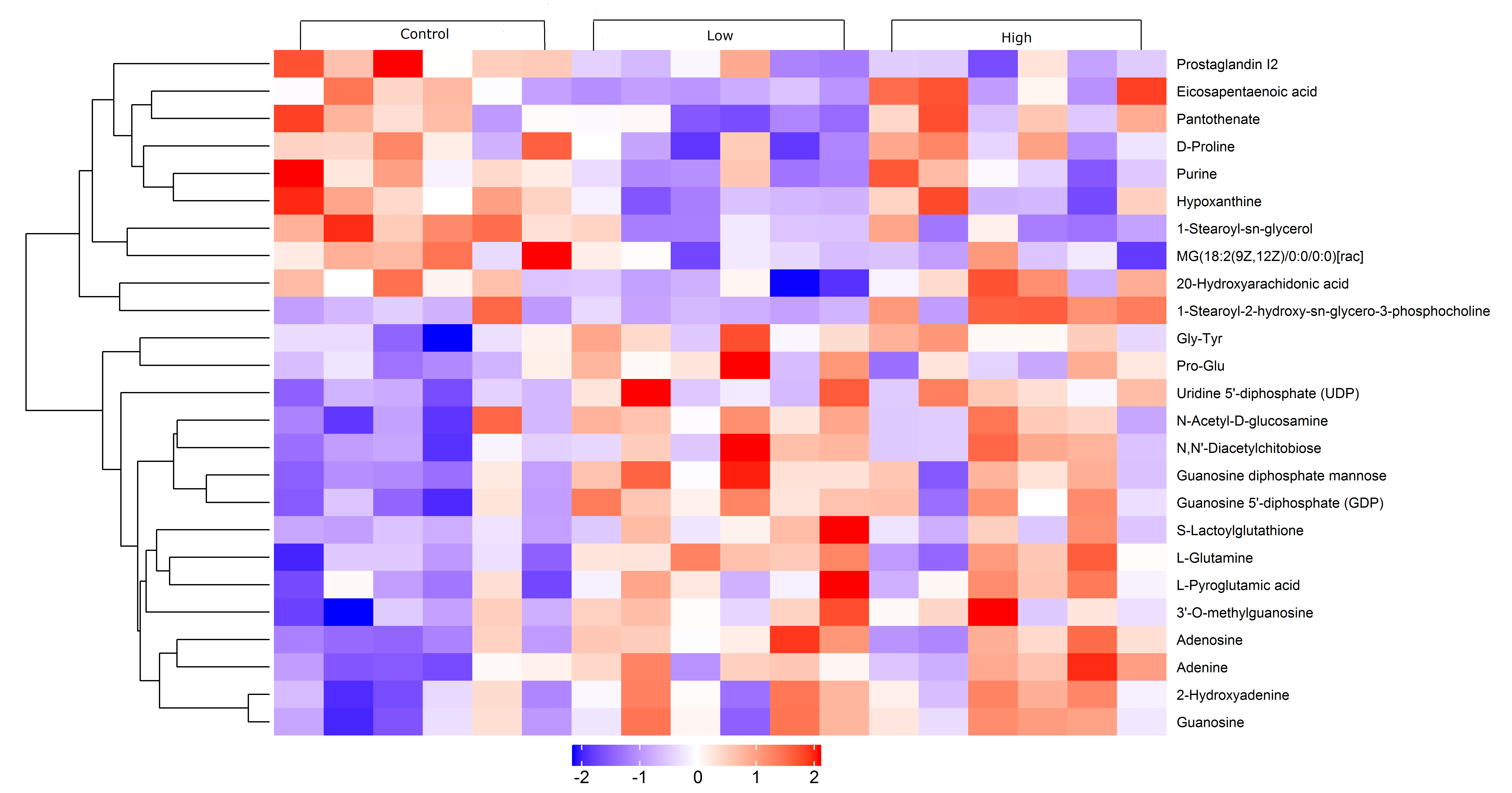


**(A)**

**
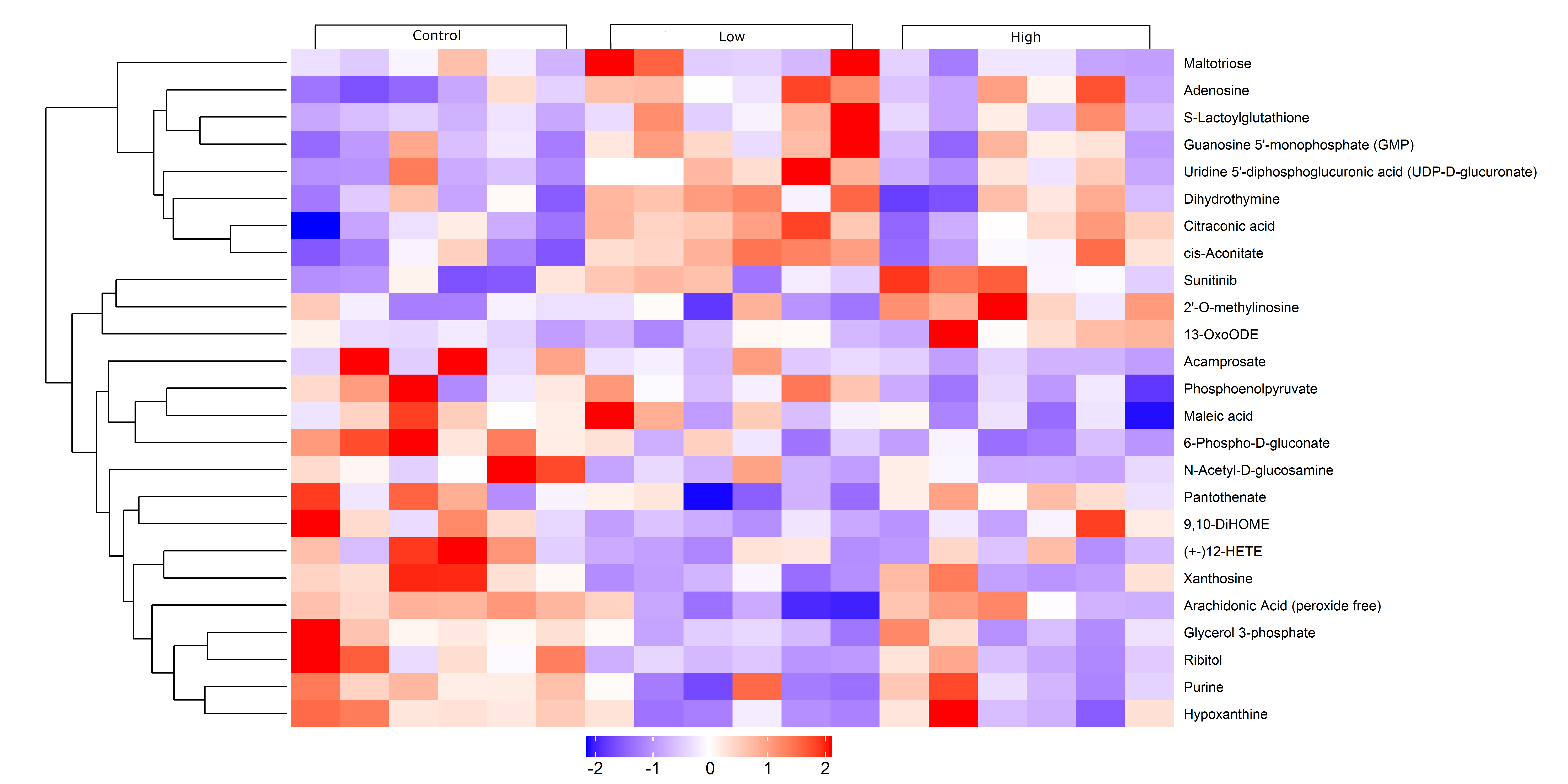
**

**(B)**

# Fig. S3. Heap maps produced by clustering of the differential metabolites in the liver using Cluster 3.0 software (http://bonsai.hgc.jp/~mdehoon/software/cluster/software. htm) coupled with Java Treeview package ([**http://www.java.com/**](http://www.java.com/)).

# (A) in positive model; (B) in negative mode.

| **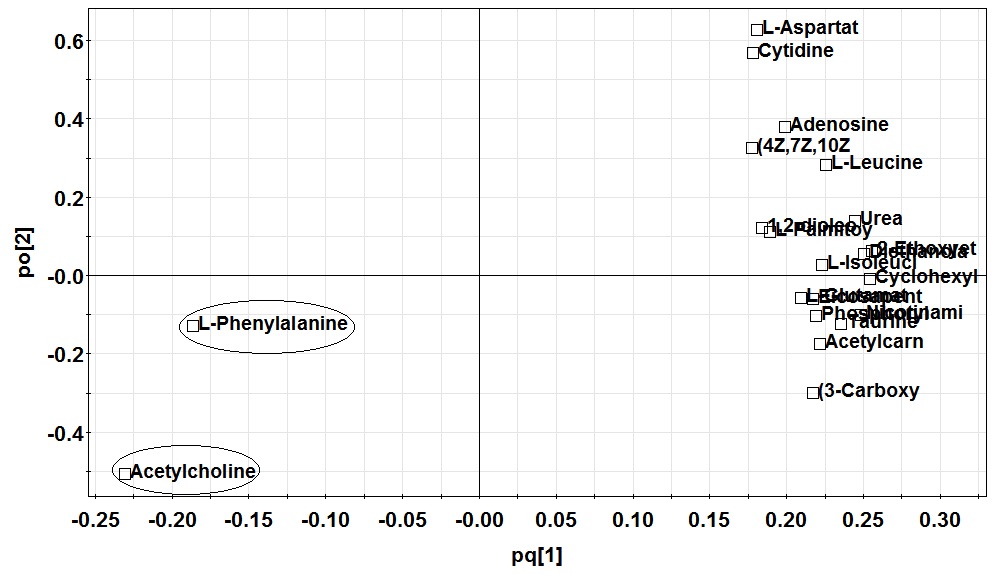**  **(A)** |
| --- |
| 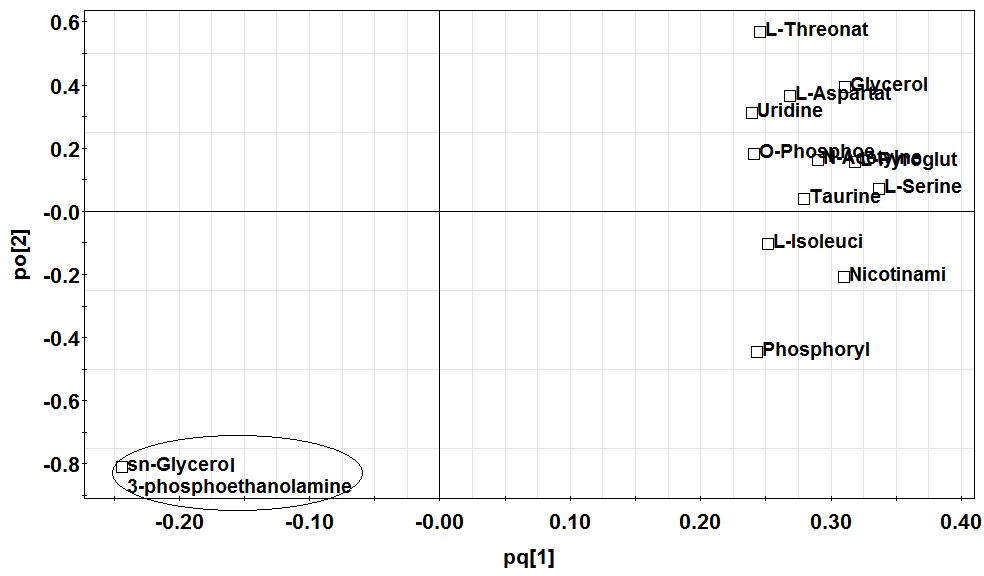  **(B)** |

Fig. S4. OPLS/O2PLS-DA loading plots in the hippocampus between the control group and the low-dose group. (A) in positive model; (B) in negative mode.

| **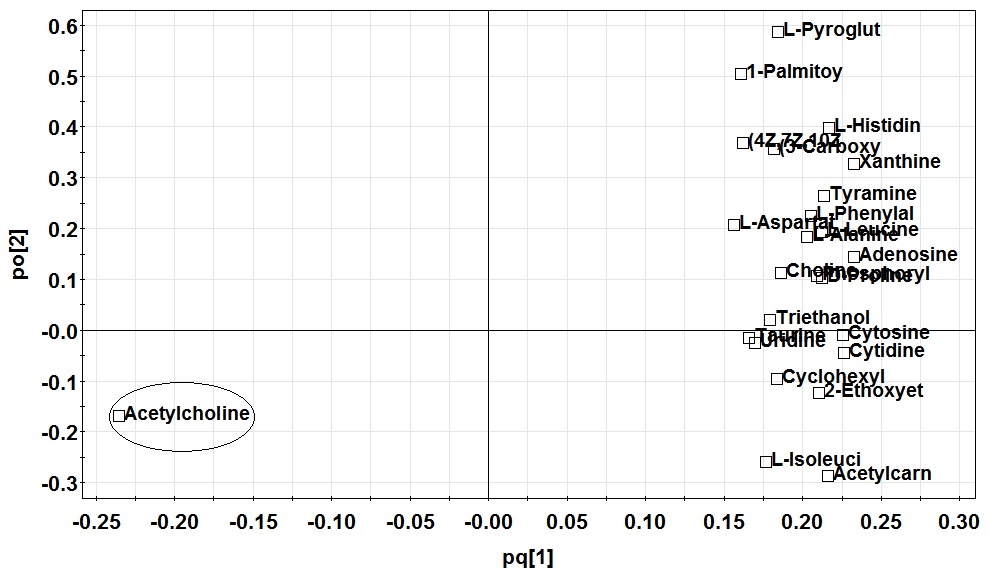**  **(A)** |
| --- |
| 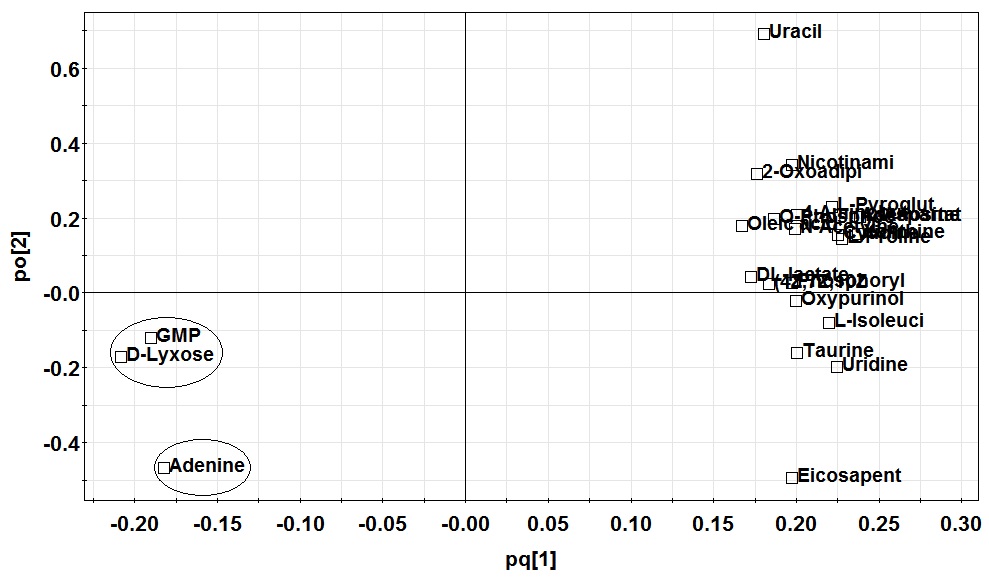  **(B)** |

Fig. S5. OPLS/O2PLS-DA loading plots in the hippocampus between the control group and the high-dose group. (A) in positive model; (B) in negative mode.

| **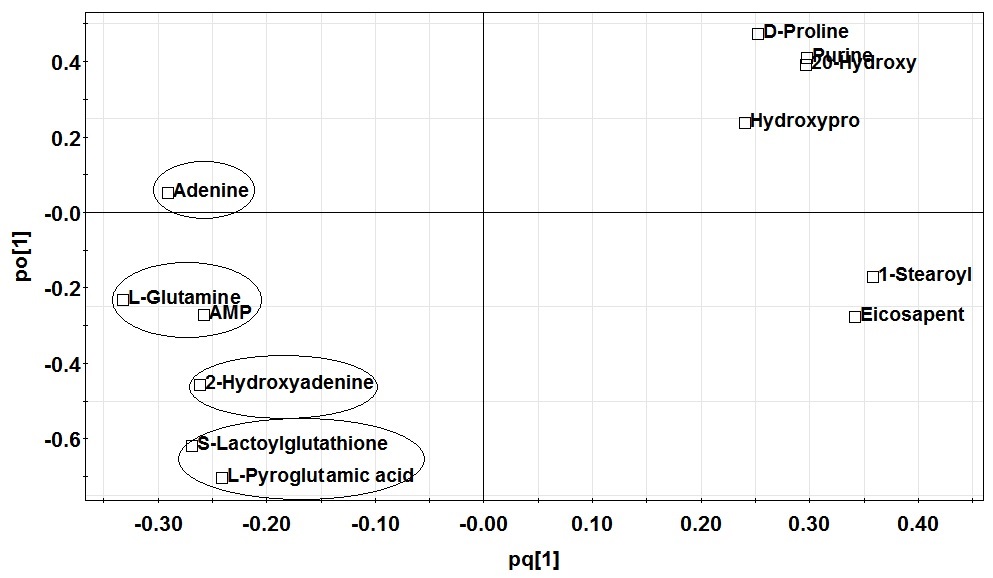**  **(A)** |
| --- |
| 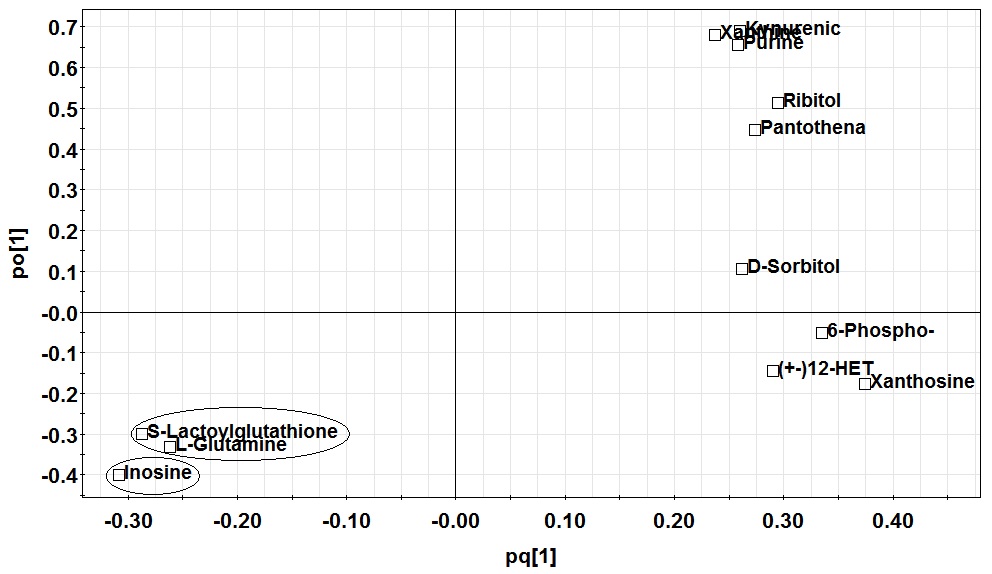  **(B)** |

Fig. S6. OPLS/O2PLS-DA loading plots in the liver between the control group and the low-dose group. (A) in positive model; (B) in negative mode.

| **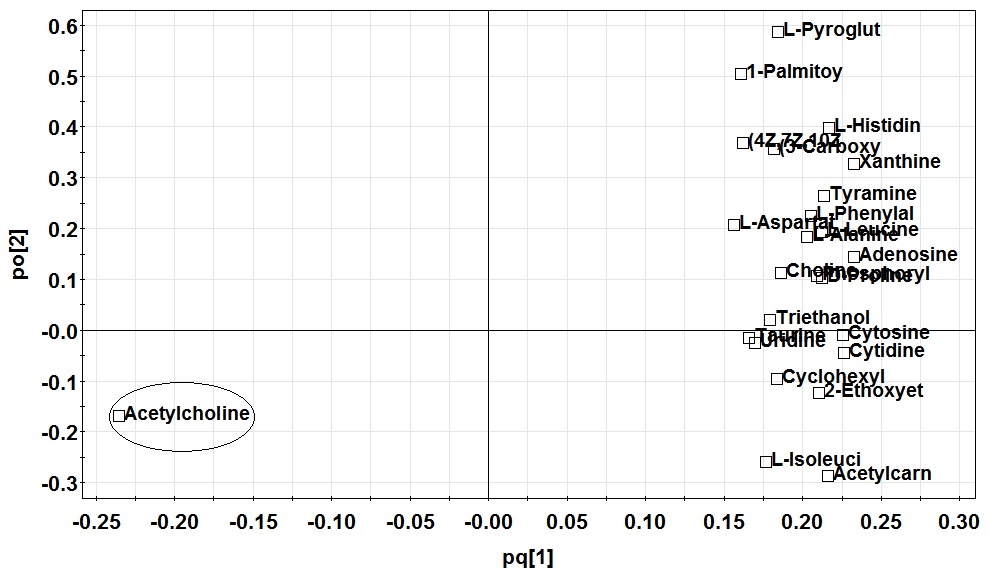**  **(A)** |
| --- |
| 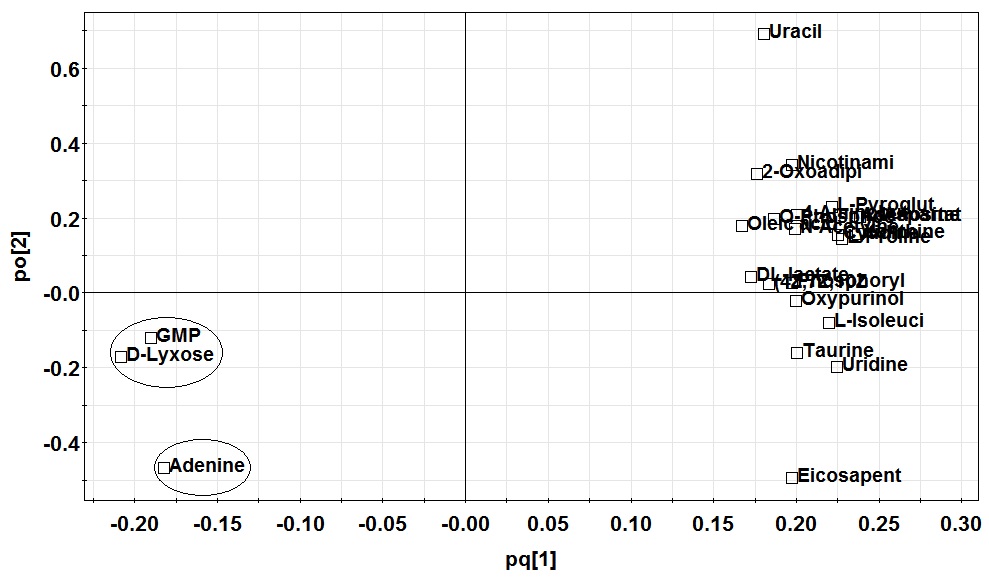  **(B)** |

Fig. S7. OPLS/O2PLS-DA loading plots in the liver between the control group and the high-dose group. (A) in positive model; (B) in negative mode.
